# Multifaceted disruption of AMPA receptor signaling by *CACNG8* variants: Integrated evidence from human genetics and molecular simulation

**DOI:** 10.1016/j.csbj.2025.09.038

**Published:** 2025-10-03

**Authors:** Simona Alibrandi, Domenico Mordà, Concetta Scimone, Giorgia Abate, Ignacio Babiloni Chust, Carmela Rinaldi, Lucia Poggi, Sergio Zaccaria Scalinci, Rosalia D’Angelo, Antonina Sidoti, Luigi Donato

**Affiliations:** a Department of Biomedical and Dental Sciences and Morphofunctional Imaging, University of Messina, Messina 98125, Italy; b Department of Biomolecular Strategies, Genetics and Cutting-Edge Therapies, I.E.ME.S.T, Palermo 90139, Italy; c Department of Veterinary Sciences, University of Messina, Messina 98122, Italy; d Department of Cellular, Computational and Integrative Biology (CIBIO), University of Trento, Trento 38123, Italy; e Department of Medical and Surgical Sciences, University of Bologna, Bologna 40121, Italy

**Keywords:** *CACNG8*, AMPA receptors, TARP γ-8, IRDs, Modifier genes, Molecular dynamics simulations, Molecular docking

## Abstract

The Transmembrane AMPA Receptor Regulatory Protein Gamma-8 (TARP γ-8), encoded by *CACNG8*, regulates AMPA receptor (AMPAR) trafficking, gating, and synaptic localization. Although well-characterized in central synapses, its role in retinal development and disease remains underexplored. This study investigates *CACNG8* within the context of inherited retinal dystrophies (IRDs) by integrating human genetics within silico structural analysis. Whole Exome Sequencing (WES) was performed on IRD-affected families with atypical phenotypes—such as residual photoreceptor activity alongside severe optic atrophy or abnormal electroretinogram (ERG) profiles—yet no biallelic mutations in known IRD genes. In all families, rare heterozygous or compound heterozygous *CACNG8* variants were identified, including a recurrent stop-gain (p.Arg123Ter) and two missense variants (p.Leu96Val, p.Val102Met), suggesting a potential modifier role. To explore their functional impact, we modeled 14 AMPAR-associated postsynaptic complexes, comparing wild-type and mutant TARP γ-8 configurations. These included auxiliary subunits (CACNG2–7, CNIH2/3), scaffolding proteins (PSD93, PSD95), and regulators (PPP3CA, RIMBPs). Docking and MM-PBSA/MM-GBSA analyses revealed that truncation or destabilization of CACNG8 severely reduced complex stability (ΔΔG > 20 kcal/mol) and altered binding geometry. Molecular dynamics simulations highlighted increased structural perturbations, reduced hydrogen bonding, and greater conformational disorder in mutant assemblies. Grid Inhomogeneous Solvation Theory (GIST) and PCA/TICA analyses further revealed diminished water structuring and constrained conformational landscapes. Altogether, our findings support the potential role of *CACNG8* as a genetic modifier in IRDs, pending further validation in larger cohorts. The study illustrates how combining genomic and structural approaches can uncover hidden contributors to complex retinal disorders.

## Highlights

• Rare *CACNG8* variants identified in IRD patients with atypical phenotypes.
• Molecular docking shows CACNG8 mutations disrupt AMPAR complex stability.
• Arg123Ter truncation leads to severe loss of PDZ-mediated interactions.
• MD simulations reveal increased conformational disorder in mutant assemblies.
• Combined in silico and genomic analysis supports *CACNG8* as an IRD modifier.

## Graphical Abstract

**Figure:**
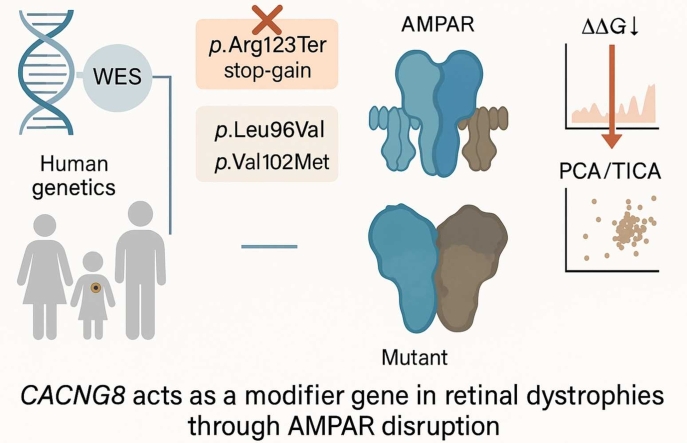

## 1 Introduction

Inherited retinal dystrophies (IRDs) represent a clinically and genetically heterogeneous group of disorders characterized by progressive photoreceptor degeneration and irreversible vision loss. These diseases encompass a broad spectrum of phenotypes ranging from retinitis pigmentosa (RP) and cone-rod dystrophy (CRD) to Leber congenital amaurosis (LCA) and macular dystrophies. To date, more than 270 genes have been implicated in IRDs, including nuclear, mitochondrial, and modifier loci, which collectively account for the significant allelic heterogeneity and phenotypic variability [1], [2]. Their inheritance patterns include autosomal dominant, autosomal recessive, X-linked, and digenic or oligogenic models, further complicating genotype-phenotype correlations [3].

Modifier genes have emerged as crucial contributors to disease expressivity and penetrance, particularly in familial cases, where canonical mutations in known IRD genes fail to fully explain the observed phenotype [4]. In this context, identifying additional genetic players is pivotal to better understand disease mechanisms and to advance personalized therapeutic strategies. Among the potential modifiers, *CACNG8* has recently gained attention for its functional relevance in central synaptic regulation and retinal neurotransmission [5].

*CACNG8* encodes the Transmembrane AMPA Receptor Regulatory Protein Gamma-8 (TARP γ-8), belongs to the claudin superfamily, and plays an essential role in AMPA receptor (AMPAR) gating, trafficking, and synaptic stabilization [6], [7]. TARPs are auxiliary subunits of AMPARs, and TARP γ-8 is particularly enriched in the hippocampus and retina, especially in retinal ganglion cells and inner nuclear layers, where it co-localizes with AMPA-type glutamate receptors [8]. Structurally, TARP γ-8 comprises four transmembrane helices, a short extracellular loop with a β-sheet-rich domain, and a cytosolic C-terminal tail containing a PDZ-binding motif that is critical for interaction with PSD95 and anchoring to postsynaptic densities [9]. This arrangement underpins its scaffolding role in regulating excitatory synaptic transmission and plasticity.

Importantly, recent structural data from cryo-EM and crystallography have demonstrated that TARP γ-8 not only modulates AMPAR gating kinetics but also stabilizes ligand-binding domain (LBD) conformations and interacts with auxiliary components such as CNIH2 and GRIA1 [7]. Several contact points between TARP extracellular β-loops and AMPAR subunits are crucial for proper receptor activity, and missense or truncating mutations in these regions may disrupt receptor assembly, trafficking, and glutamate-induced signaling.

Our study stems from a multifamilial genetic screening project focused on unexplained or atypical IRD cases. Whole-exome sequencing (WES) in multiple probands revealed combinations of pathogenic or likely pathogenic variants in known IRD genes together with rare, deleterious mutations in *CACNG8*, notably the nonsense variant p.Arg123Ter and the missense variants p.Leu96Val, p.Val102Met, and p.Val146Gly. These findings, supported by the segregation analysis, suggest that *CACNG8* may act as a modifier gene, influencing disease severity or phenotypic onset in genetically complex IRDs [10].

To investigate this hypothesis, we employed an integrated multi-tiered strategy combining:

• Human exome data and in silico pathogenicity prediction pipelines;
• Molecular docking and molecular dynamics (MD) simulations to assess the structural and functional consequences of *CACNG8* mutations on AMPAR-related synaptic architecture;
• Post-simulation analysis, including solvation energetics (GIST), principal component analysis (PCA/TICA), residue-based flexibility (RMSF), and MM/PBSA free energy estimates.

This interdisciplinary framework allowed us to explore how deleterious mutations in *CACNG8* may compromise AMPAR function and synaptic stability, offering a mechanistic explanation for phenotypic heterogeneity in IRDs. Our findings advance the understanding of *CACNG8* as a critical regulator at the intersection of retinal synaptic integrity and genetic modulation, highlighting its potential as a novel diagnostic and therapeutic target in IRD pathology.

## 2 Material and methods

### 2.1 Human molecular genetic analysis

This study enrolled affected individuals and family members from multiple unrelated pedigrees presenting with clinical features compatible with IRDs, primarily within the CRD/RCD (Rod-Cone Dystrophy) spectrum. Written informed consent was obtained from all participants or legal guardians, in accordance with the Declaration of Helsinki and under the approval of the Scientific Ethics Committee of the Azienda Ospedaliera Universitaria – Policlinico “G. Martino” Messina.

Comprehensive clinical evaluation was performed on all enrolled individuals, including best-corrected visual acuity (BCVA), fundus examination, color fundus photography, spectral-domain optical coherence tomography (SD-OCT), full-field electroretinography (ff-ERG), pattern visual evoked potentials (PEV), and Goldmann kinetic perimetry. Phenotypic variability was carefully documented across family members, with a focus on optic nerve atrophy, residual cone and rod responses, and atypical progression patterns diverging from canonical IRD classifications.

Peripheral blood samples (10 mL) were collected in EDTA-coated tubes. Genomic DNA was extracted using the QIAamp DNA Blood Mini Kit (Qiagen, Hilden, Germany), and its quality was verified via NanoDrop 2000 spectrophotometry (Thermo Fisher Scientific), while DNA integrity was assessed through 0.8 % agarose gel electrophoresis.

Whole-exome sequencing (WES) was performed on 18 individuals across six unrelated families, including 6 probands, 8 affected relatives, and 4 unaffected relatives, to enable segregation analysis. Exonic libraries were prepared using the SureSelect Human All Exon v7 kit (Agilent Technologies), which targets approximately 51 Mb (∼19,400 genes), and sequenced on an Illumina NovaSeq 6000 platform to obtain paired-end 150 bp reads. The average sequencing depth was 112 × , with > 97.6 % of exonic bases covered at ≥ 20 × and > 99.4 % at ≥ 10 × . Quality trimming was performed using Trimmomatic v0.39 (parameters: LEADING:3, TRAILING:3, SLIDINGWINDOW:4:15, MINLEN:36). Sequence data were processed using a validated GATK-based pipeline [11]. Reads quality was assessed using FastQC v0.12 [12] and filtered by Trimmomatic v0.39 [13]. The average proportion of reads removed after trimming was below 3 %, indicating limited impact on total sequencing yield. Reads were aligned to the GRCh38/hg38 reference genome using BWA-MEM v0.7.19 [14], with an average unique alignment rate of 96.2 % across all samples.

Duplicate reads were marked with MarkDuplicated v4.0.1.1, and base quality score recalibration along with indel realignment was carried out using GATK v4.6.2.0. Variant calling was conducted using HaplotypeCaller, and VCFs were normalized using bcftools v1.21 [15]. Post-calling quality control included inspection of read depth, genotype quality (GQ), and variant quality (QUAL) metrics. Variants with low depth (<10 ×) or low confidence (QUAL < 30) were excluded prior to downstream annotation and prioritization.

Variant annotation included functional prediction with ANNOVAR [16], Ensembl VEP [17], and SnpEff [18]; population frequency comparison against gnomAD v4.1 [19], 1000 Genomes [20], and ESP6500 [21]; and *in silico* pathogenicity scoring using SIFT [22], PolyPhen-2 [23], MutationTaster [24], and CADD [25]. Evolutionary conservation was assessed via PhyloP and PhastCons [26]. Synonymous variants were excluded unless predicted to affect splicing (dbscSNV > 0.6). Retained variants were exonic or splicing, rare (MAF < 0.01), predicted deleterious by at least two tools, and not annotated as benign in ClinVar or LOVD. Additional filtering was based on CADD > 20, SIFT = deleterious, and PolyPhen-2 = probably damaging.

For each proband, causative mutations in known IRD-associated genes (as curated in RetNet, [27]) were first examined. In families where the observed phenotype was not fully explained by known pathogenic variants or demonstrated incomplete segregation, we hypothesized the presence of modifier genes. Candidate modifiers were prioritized using phenotype-driven tools such as Aerts et al. and ToppGene [28], and further explored using STRING [29] for protein–protein interaction (PPI) networks, GeneMANIA [30] for co-expression and functional linkage, and DAVID/Enrichr [31], [32] for Gene Ontology and KEGG enrichment analyses. STRING v11.5 was used with a minimum required interaction score of 0.7 (high confidence). A list of 38 candidate genes was submitted as input, and 26 were found to have direct or indirect interactions. The network included experimentally validated interactions, database-curated links, and co-expression evidence. Interactions were prioritized based on physical evidence and connectivity within the core subnetwork. GeneMANIA analysis was conducted using default settings, with all data sources enabled (co-expression, physical interactions, genetic interactions, pathways, co-localization, and shared protein domains). The input set included the same 38 candidate genes used in STRING. The resulting network highlighted genes with shared pathways and high co-expression with known IRD genes, supporting functional convergence. TSS-proximal variants (±1 kb) were annotated using RegulomeDB and ENCODE chromatin state data.

Final gene prioritization was performed by integrating STRING and GeneMANIA network scores with deleteriousness and phenotype-ranking outputs. The gene *CACNG8* consistently emerged among the top five candidates across this consensus framework. Interactions supported by experimental or curated database evidence (as annotated in STRING) were prioritized over predicted or text-mined associations. Only high-confidence nodes supported by both STRING and GeneMANIA were retained in the consensus gene list, which was then integrated with variant pathogenicity scores and phenotype relevance. A full overview of the prioritization workflow is shown in Supplementary Figure 9.

Starting from 1317 rare and predicted-deleterious variants (across 18 samples), we identified 237 unique genes not previously linked to IRDs in RetNet. These were analyzed via STRING and GeneMANIA and further subjected to functional enrichment using GO and KEGG through DAVID and Enrichr. Integration of network connectivity, phenotypic relevance (via ToppGene), and variant-level deleteriousness yielded a consensus list of top five candidate modifier genes, among which *CACNG8* consistently ranked first due to its strong network centrality, known retinal expression, and co-occurrence with pathogenic IRD mutations.

Across different families, the strongest candidate emerging from this integrative pipeline was *CACNG8*, the gene encoding the TARP γ-8 auxiliary subunit of AMPA receptors. Multiple rare and likely deleterious variants were identified in this gene, including the nonsense variant c.367 C>T (p.Arg123Ter), the missense variant c.436 C>T (p.Leu146Val), and the previously unreported c.286 C>T (p.Leu96Val), which was found in homozygous or compound heterozygous state alongside pathogenic mutations in core IRD genes such as *GRIA1*, *CNGB3*, and *PDE6B*. Notably, segregation patterns in these families were consistent with the hypothesis of a modifier role for *CACNG8*, especially in individuals carrying digenic or multigenic combinations.

Notably, post-filtering analysis revealed that high-impact variants in *CACNG8* (such as the nonsense p.Arg123Ter and compound heterozygous combinations) were consistently observed in affected individuals, but absent or heterozygous-only in unaffected relatives. This segregation pattern further supports a potential modulatory role for *CACNG8* in modifying IRD severity.

The variants of interest were validated by Sanger sequencing on an ABI 3500 Genetic Analyzer (Thermo Fisher Scientific), using primers designed with Primer3 [33]. Bidirectional sequencing data were aligned and interpreted using SnapGene Viewer (www.snapgene.com), and segregation analyses were conducted within available pedigrees.

Finally, structural predictions of the impact of these variants were conducted using SwissModel [34], YASARA [35], and PyMOL (The PyMOL Molecular Graphics System, v3.0 Schrödinger, LLC) for 3D modeling, and thermodynamic stability estimations were computed with FoldX [36] and mCSM [37]. SwissModel homology modeling was performed using the web server (release 2023–11), with default settings for model building and QMEAN scoring. SwissModel was used in automated mode, and only templates with sequence identity ≥ 30 % and alignment coverage > 70 % were retained. Models with GMQE scores < 0.6 were excluded. The best-scoring template for CACNG8 had 35.2 % identity and 82 % coverage, with a QMEANDisCo global score of 0.66. YASARA Structure v22.9.24 was used to refine geometry and optimize hydrogen bonding using the YAMBER3 force field, with minor adjustment of torsion angles to minimize steric clashes. PyMOL v3.0 was used for visualization and structural inspection. FoldX v5.0 was run with the RepairPDB function prior to ΔΔG calculations. No non-default parameters were applied in FoldX or mCSM. The stop mutation p.Arg123Ter was predicted to truncate key transmembrane helices and cytosolic PDZ-binding motifs, while Leu146Val and Val102Met affected regions essential for AMPAR interaction. These in silico assessments supported the hypothesis of functional disruption and laid the foundation for subsequent docking and dynamics studies.

Full genotypic information, including zygosity, inheritance models, segregation patterns, and gnomAD allele frequencies of *CACNG8* variants across all WES samples, is provided in Supplementary Table S2.

### 2.2 Molecular docking and molecular dynamics simulations

#### 2.2.1 Ligand and receptor preparation

To investigate the molecular mechanisms underlying the impact of *CACNG8* variants on AMPA receptor signaling, an accurate preparation of protein receptors and ligand structures was carried out prior to docking simulations. The structural models of all protein components involved—TARP γ-8 (wild-type and mutant forms), AMPA receptor subunits (GRIA1–GRIA4), PSD95, CNIH2, and associated interactors (e.g., PPP3CA, CACNG2)—were obtained from multiple sources.

Where available, experimentally determined structures were retrieved from the Protein Data Bank (PDB, https://www.rcsb.org). For proteins lacking high-resolution structures, homology models were generated using SwissModel and further refined using YASARA. Mutant variants of *CACNG8* were created by site-directed *in silico* mutagenesis using PyMOL, including:

• p.Leu96Val (hypomorphic variant)
• p.Val102Met
• p.Val146Gly
• p.Arg123Ter (premature stop codon leading to truncation)

These substitutions were introduced using PyMOL’s mutagenesis wizard, followed by local minimization and loop rebuilding where truncations altered secondary structures. Final models were validated using MolProbity [38], PROCHECK [39], and ProSA-web [40], with Ramachandran outliers below 2 % and Z-scores within the experimentally expected range.

For homology-modeled proteins, sequence regions lacking reliable template coverage were carefully managed. Segments with low-quality predictions—either due to poor sequence identity or alignment gaps—were rebuilt using MODELLER v10.4 or replaced by high-confidence AlphaFold2-derived structures, retrieved from the AlphaFold Protein Structure Database. In cases of long unstructured loops or terminal extensions, regions predicted as intrinsically disordered were truncated or masked to avoid artificial flexibility. Final models included only structurally coherent domains, confirmed through MolProbity and ProSA-web assessments, to ensure robustness in docking and molecular dynamics simulations.

The glutamate ligand (PubChem CID: 611) was downloaded in SDF format and converted to MOL2 and PDB formats using Open Babel v3.1.1 [41]. It was modeled in its physiologically relevant zwitterionic form, reflecting the dominant species at pH 7.4. Specifically, both carboxyl groups were deprotonated (–COO⁻), while the amino group was protonated (–NH₃⁺), resulting in a net formal charge of –1. This state was preserved throughout all stages of docking and molecular dynamics simulations. Ligand structures were further processed in Avogadro v.1.2.0 [42] using the MMFF94s force field and optimized with YASARA PM3 +ESP minimization. Multiple low-energy conformers were generated and manually validated. All ligand files used in docking (PDBQT format) were visually and computationally verified to ensure correct protonation and charge assignment. All generated glutamate conformers were additionally inspected and validated for structural quality. We manually evaluated bond lengths, bond angles, torsion geometries, and potential steric clashes using Avogadro and PyMOL. Bond lengths and angles were evaluated against idealized geometries as defined in the Protein Geometry Database. Torsion angles were checked using Ramachandran plots, with < 2 % residues allowed in disallowed regions. Steric clashes were flagged in Avogadro if Van der Waals overlaps exceeded 0.4 Å. All models were inspected independently by two experienced structural biologists to ensure consistency, and only conformers passing all criteria were retained for energy minimization. Structures exhibiting any geometrical abnormalities, such as eclipsed conformations or distorted ring penetrations, were excluded. Final conformers were further checked with MolProbity to ensure compatibility with standard chemical geometries. Only structurally sound, low-energy conformers were retained for all subsequent simulations. Structurally sound conformers were defined as those with no major backbone distortion, Ramachandran outliers < 2 %, and no steric clashes exceeding a Van der Waals overlap of 0.4 Å. Low-energy conformers were defined as those showing minimized energy states following FoldX RepairPDB correction and ΔΔG within the range expected for tolerated substitutions (<2 kcal/mol, when applicable). Only conformers satisfying both structural and energetic criteria were retained for further docking and MD simulations.

Metal ions were treated with attention to physiological relevance:

• Ca²⁺ was modeled with full octahedral coordination, using topology templates from CHARMM36 and AmberTools libraries [43]
• Na⁺ and K⁺ were parameterized as monovalent cations with partial hydration spheres [44]

All ions were assigned formal charges (+2 for Ca²⁺, +1 for Na⁺/K⁺), and converted to PDBQT via MGLTools 1.5.7 [45], with zero torsional degrees of freedom.

Ligand and protein structures were preprocessed and formatted using a hybrid Open Babel + AutoDockTools pipeline. For ligands, Gasteiger partial charges were computed, rotatable bonds were defined (except for ions), and multiple conformers were generated where applicable. Proteins were protonated at physiological pH, with polar hydrogens added and non-polar hydrogens merged, according to AutoDock conventions.

Custom ligand libraries were compiled in.pdbqt format, comprising 50 + glutamate conformers (zwitterionic at pH 7.4) and full parameterization of monatomic and hydrated Ca²⁺, Na⁺, and K⁺ ions.

Binding site definition was informed by a dual strategy:

• Literature-informed selection of key motifs in *CACNG8*: particularly the β1–β2 extracellular loop and the C-terminal PDZ-binding region [46]
• *In silico* prediction of potential cavities using COACH-D [47], PrankWeb [48], and SiteMap (Schrödinger, LLC, https://www.schrodinger.com/platform/products/sitemap/)

Receptor grids were manually defined to encompass both canonical and non-canonical pockets, with dimensions ranging from 24 × 24 × 24 Å to 36 × 36 × 36 Å, centered on the interfacial zones of the CACNG8–GRIA1/PSD95/CNIH2 assemblies.

Interfacial residues involved in docking were identified and annotated using PDBePISA [49], including:

• Residues involved in hydrogen bonding and salt bridge formation
• Aromatic/hydrophobic clusters (e.g., Phe, Tyr, Leu hotspots)
• Known AMPAR contact points validated via cryo-EM

In total, 14 structural assemblies were modeled—each reflecting a specific *CACNG8* mutant (or wild-type) in complex with relevant AMPAR subunits and scaffold proteins—providing a robust and validated dataset for downstream molecular docking and dynamics simulations.

#### 2.2.2 Docking protocols and scoring strategies

Following the structural preparation of ligands and receptors, molecular docking simulations were carried out to predict the binding affinity and pose stability of glutamate and key ions (Ca²⁺, Na⁺, K⁺) within each of the 14 CACNG8-based complexes. Docking protocols were designed to account for both ligand–protein and protein–protein interactions, including multimeric assembly behavior when appropriate.

For all ligand–protein docking experiments (e.g., glutamate to TARP–AMPA complexes), we used AutoDock Vina v1.2.7 [50] and AutoDock 4.2.6 [45], allowing cross-validation between scoring functions and sampling algorithms. Vina was preferred for its speed and global pose prediction, while AutoDock4 enabled deeper analysis of electrostatic complementarity and desolvation contributions.

Each docking grid was centered on either:

• The β1–β2 loop of CACNG8 (residues 58–73), where ligand binding may allosterically modulate AMPA function;
• Or the C-terminal tail (residues 404–423), critical for PDZ-domain interaction with PSD95.

Grid box sizes ranged from 24 × 24 × 24 Å (for focused ligand binding studies) to 40 × 40 × 40 Å (for extended interfacial searches), with exhaustiveness set to 32 in Vina, and genetic algorithm (GA) runs set to 250 in AutoDock4. Ten best poses were retained per ligand, with clustering performed using an RMSD cutoff of 2.0 Å.

All Vina docking outputs were rescored using AutoDock4’s semi-empirical free energy function, and post-filtered based on:

• Binding affinity thresholds (< –6.0 kcal/mol considered significant)
• RMSD from reference poses (< 2.5 Å for stable clustering)
• Buried surface area (BSA) (> 800 Å² for protein–protein complexes)
• Hydrogen bonding networks and salt bridge formation, as calculated using PLIP [51] and PoseView [52]

Protein–protein docking, used to validate CACNG8 interaction with GRIA1, PSD95, and CNIH2, was performed using:

• HADDOCK2.4 [53]
• ClusPro v2.0 [54]
• ZDOCK v3.0.2 [55]

Each run used experimentally informed or literature-based interaction residues as active or passive restraints. Interface residues from cryo-EM structures were employed as anchor points when available (e.g., AMPAR LBD for TARP β1–β2 loop; PSD95 PDZ domain for CACNG8 C-tail). HADDOCK scoring incorporated van der Waals, electrostatics, desolvation, and buried surface area. Clustering was performed by interface RMSD, retaining the top 5 clusters for evaluation.

To ensure biological plausibility, all docking poses were visually inspected and filtered based on structural context. Poses located in transmembrane helices or deeply buried hydrophobic regions—lacking experimental support or known functional relevance—were excluded from further analysis, even when associated with favorable docking scores. Only poses consistent with literature-reported extracellular interaction zones (e.g., β1–β2 loop, PDZ-binding C-tail) and solvent-accessible interfaces were retained for subsequent MD simulations and energetic evaluations.

To prioritize biologically plausible poses, a multi-tier filtering pipeline was applied:

• Docking energy (Vina < –7.5 kcal/mol; HADDOCK score < –90)
• Pose reproducibility (≥3 conformers within a 2 Å RMSD cluster)
• Conformational stability (evaluated post hoc by short MD validation)
• Interface complementarity and clash score (via PDBePISA and MolProbity)

In select cases, custom grid maps were generated in AutoGrid4 to include flexible side chains at the binding interface (e.g., Glu60, Arg404), allowing local induced fit. These enhanced protocols were particularly important in mutants where the interface was structurally altered (e.g., Arg123Ter truncation, Val146Gly shifts).

Finally, results from all 14 complexes—including wild-type and mutant variants—were cataloged in a docking matrix summarizing:

• Best predicted binding affinity (kcal/mol)
• Pose RMSD
• Interfacial area
• H-bond/salt bridge count
• Receptor–ligand contact fingerprint (IFP) profiles

This matrix was subsequently used as the starting point for Molecular Dynamics simulations, and for comparative interpretation across mutant classes, allowing robust correlation with phenotypic and functional predictions.

#### 2.2.3 Molecular dynamics simulation protocols

To complement molecular docking predictions and evaluate the dynamic behavior of TARP γ-8–AMPA receptor complexes in the presence of glutamate and relevant ionic species, atomistic molecular dynamics (MD) simulations were conducted using GROMACS 2025.0 [56]. All systems corresponded to the 14 previously constructed ternary or quaternary complexes, including both wild-type and mutated CACNG8 variants.

All protein structures were processed using the AMBER99SB-ILDN force field, while the ligand (glutamate) was parameterized using the General Amber Force Field (GAFF) with partial charges computed via the AM1-BCC method using *Antechamber* [57]. Although the OPC water model has been primarily benchmarked in combination with newer AMBER force fields (e.g., ff14SB, ff19SB), we employed it here with AMBER99SB-ILDN to maintain consistency with IRD-related studies and validated its use through post hoc dynamic stability metrics. Ion topologies for Ca²⁺, Na⁺, and K⁺ were derived from the Joung and Cheatham parameters [44] and included in physiological proportions depending on the modeled neuronal environment (e.g., extracellular vs cytosolic compartments).

Each complex was placed at the center of a triclinic dodecahedral box with 1.0 nm padding, solvated using the OPC water model [58], and neutralized with Na⁺ or Cl⁻ ions to achieve an ionic strength of 0.15 M. Energy minimization was performed using the steepest descent algorithm until the system reached a force convergence threshold of < 1000 kJ/mol/nm.

The system was equilibrated in two steps:

• NVT ensemble (constant Number, Volume, Temperature) for 100 ps, with position restraints on heavy atoms, using a V-rescale thermostat at 310 K.
• NPT ensemble (constant Number, Pressure, Temperature) for 500 ps, applying isotropic pressure coupling via the Parrinello-Rahman barostat at 1 bar.

Each production run was carried out for 100 ns, using a 2 fs timestep and LINCS constraints on all hydrogen-containing bonds. The Particle Mesh Ewald (PME) method was applied for long-range electrostatics, with a cutoff of 1.0 nm for both Coulombic and van der Waals interactions.

Trajectory outputs were saved every 10 ps, generating 10,000 frames per simulation, totaling 1.4 µs across all complexes. The simulations were performed on a high-performance Mac Studio equipped with an Apple M2 Ultra chip (24-core CPU, 76-core GPU) and 192 GB of unified memory, leveraging GPU acceleration via Metal.

System stability was monitored through the evolution of total energy, temperature, pressure, and density, extracted from.edr and.log files. No major instabilities or system crashes were detected. Representative frames were extracted every 10 ns for post-processing analyses using MDAnalysis v2.3.0 [59] and VMD v1.9.4a [60], forming the basis for structural analysis, interaction fingerprinting, and solvation mapping.

Simulation data provided the structural basis for:

• Root Mean Square Deviation (RMSD) and Root Mean Square Fluctuation (RMSF) via gmx rms and gmx rmsf
• Hydrogen bond persistence via gmx hbond
• Solvent-accessible surface area (SASA) with gmx sasa
• Radial distribution functions (RDF) with gmx rdf, focusing on glutamate and ions near PDZ and β1–β2 loop regions
• Principal component analysis (PCA) and Time-lagged independent component analysis (TICA) with gmx covar, gmx anaeig, and PyEMMA [61]
• GROMOS clustering (gmx cluster, cutoff = 0.2 nm) to determine dominant conformational states
• MM-PBSA and MM-GBSA energy decomposition using gmx_MMPBSA v1.6.2 [62]
• Grid Inhomogeneous Solvation Theory (GIST) mapping

All simulations and subsequent analyses followed standard validation protocols. Where appropriate, multiple independent replicates (n = 3) were run to assess convergence and reproducibility of the results.

Trajectory videos were generated as qualitative illustrations of the simulation systems using default rendering settings in PyMOL and VMD. To maintain clarity and reduce visual clutter, elements such as chain-specific color coding, orientation standardization, and explicit labeling were minimized. However, all structural and energetic analyses—including RMSD, RMSF, hydrogen bonding, ligand positioning, and interfacial metrics—were conducted on validated atomic trajectories, independent of video rendering. Apparent visual simplifications do not affect the scientific interpretation or conclusions of the study. This clarification has been added in response to reviewer feedback. To facilitate reproducibility, we now provide full details of simulation protocols, including force field choices, topology generation, solvation and box size, ionic strength (0.15 M NaCl), time step (2 fs), equilibration phases (100 ps NVT, 500 ps NPT), and production settings (PME electrostatics, LINCS constraints). These parameters are summarized in Supplementary Table S1, which also reports energy decomposition settings for MM-PBSA, MM-GBSA, and GIST solvation analysis.

#### 2.2.4 Free energy and solvation analysis

To elucidate the energetic contributions of *CACNG8* mutations in AMPAR-containing assemblies, we employed a dual analytical strategy combining Molecular Mechanics Poisson–Boltzmann Surface Area (MM-PBSA) and Grid Inhomogeneous Solvation Theory (GIST). This integrated approach enabled both global free energy estimation and spatially resolved solvation mapping.

MM-PBSA and MM-GBSA calculations were carried out using gmx_MMPBSA v1.6.2 interfaced with GROMACS 2025.0. Energy decomposition included van der Waals, electrostatic, polar and non-polar solvation terms, computed over 250 evenly spaced frames from the final 50 ns of production trajectories. Although entropy terms (TΔS) were not included due to convergence limitations, the enthalpic and solvation components allowed for relative comparisons across complexes. These ΔΔG estimates should be interpreted qualitatively rather than as precise quantitative measures, as absolute binding free energies may be biased in the absence of full entropic contribution.

Both MM-PBSA and MM-GBSA methods were applied to the same simulation frames using gmx_MMPBSA v1.6.2. The solute dielectric constant was set to 4.0, following widely accepted values for protein interiors to better reflect electrostatic screening in a polar environment. Although the absolute free energy values differed slightly between the PB and GB approaches, both yielded consistent trends in relative ΔΔG values across wild-type and mutant complexes. The MM-PBSA results are presented in the main text, while the corresponding MM-GBSA data are included in Supplementary Table S4.

In parallel, GIST analysis was performed using GIST as implemented in AmberTools23 + PyGIST on 100 representative frames per system (1 per ns), applying a 0.5 Å voxel grid surrounding each protein–ligand interface. Solvent was modeled with the OPC water model, capturing both dipolar and quadrupolar contributions. Grid analysis yielded enthalpic maps, solvent entropy (translational and orientational), and local water densities.

Structured hydration layers were conserved in Leu96Val and wild-type systems, while truncating mutations (e.g., Arg123Ter) exhibited pronounced desolvation near the C-terminal PDZ-binding domain. Intermediate behaviors were observed in Val102Met and Val146Gly variants, correlating with partial disruption of interfacial solvation and binding geometry. A detailed summary of the computational parameters used for MM-PBSA, MM-GBSA, and GIST solvation analyses is provided in Supplementary Table S1.

#### 2.2.5 Principal component analysis, time-lagged independent component analysis, and conformational clustering

To investigate the global conformational landscape and collective dynamics of each protein complex during molecular dynamics simulations, we performed a combined approach based on Principal Component Analysis (PCA) and Time-Lagged Independent Component Analysis (TICA). These techniques allowed us to reduce the high-dimensional coordinate space into a set of dominant motions, thereby facilitating the identification of relevant conformational transitions and metastable states.

Prior to dimensionality reduction, each trajectory was preprocessed by fitting atomic coordinates to the reference structure using least-squares superposition on the Cα atoms of the backbone. For PCA, the positional covariance matrix was computed using GROMACS (gmx covar), and eigenvalue decomposition was applied to extract the principal components (PCs). The first few eigenvectors, typically accounting for more than 70 % of the total atomic fluctuation, were selected to define the essential subspace and characterize dominant collective motions. A representative scree plot showing the variance explained by the top eigenvectors is reported in Supplementary Figure S9. Trajectory projections onto these PCs were visualized using GROMACS tools (gmx anaeig) and custom Python scripts, enabling a comparative inspection of wild-type and mutant systems.

TICA was employed to identify kinetically slow processes that are not necessarily associated with large structural displacements. Using the PyEMMA package v.2.5.14, TICA analysis was carried out with a lag time of 10 ns. This approach provided insight into the slowest dynamic degrees of freedom, which were particularly informative in distinguishing transition states and low-energy basins in systems harboring truncating or missense mutations.

To identify conformational states and extract representative structures, we applied a clustering algorithm based on the GROMOS method, using RMSD matrices calculated over the Cα atoms. A cutoff value of 0.2 nm was used to define clusters, and the most populated cluster was considered the dominant conformational state for each system. Representative frames were extracted from the centroid of these clusters and used for subsequent analyses, including interaction fingerprinting, pocket volume characterization, and visual inspection of structural rearrangements.

Altogether, the combination of PCA, TICA, and clustering enabled us to deconvolute both the large-scale and subtle dynamic rearrangements induced by *CACNG8* variants. This integrative approach was critical to identify the most functionally relevant conformers and assess the impact of single or combined mutations on the allosteric and binding properties of the complexes.

#### 2.2.6 Pocket characterization and interaction fingerprint analysis

To further explore how *CACNG8* mutations affect ligand binding and allosteric modulation at the molecular level, we conducted a systematic analysis of binding pocket geometry and dynamic interaction fingerprints throughout the simulation trajectories. These analyses allowed us to correlate structural changes and dynamic fluctuations with potential functional consequences on AMPAR complex regulation.

Binding site architecture was first characterized using both POCASA v1.1 [63] and KVFinder v1.2.0 [64] to quantify geometric descriptors such as pocket volume, depth, and surface curvature. Snapshots were extracted every 10 ns from each 100 ns molecular dynamics trajectory, allowing for a time-resolved profiling of cavity fluctuations. The glutamate binding site and adjacent modulatory interfaces were the primary focus of this analysis, particularly the extracellular β1–β2 loop and the C-terminal PDZ-interacting region of TARP γ-8. These regions are known to participate in essential contacts with AMPA receptor ligand-binding domains and synaptic scaffold proteins such as PSD95.

Each identified pocket was evaluated for dynamic stability by tracking its occupancy over time—specifically monitoring the frequency and persistence of glutamate positioning within the cavity, as well as the displacement or occlusion by mutated side chains. Pocket plasticity was quantified across variants to identify conformational shifts potentially responsible for altered ligand affinity or signaling efficacy.

Complementary to geometric assessment, a full interaction fingerprint (IFP) matrix was computed using PLIP v2.1.3 and extended with the MD-IFP toolset, which enables the extraction of interaction types from MD frames. These included hydrogen bonds, hydrophobic contacts, π–π stacking, cation–π interactions, and salt bridges. Residue-level contact maps were generated to visualize the temporal evolution of key interactions in both wild-type and mutant complexes.

Comparison of IFP matrices across variants revealed mutation-specific perturbations in contact profiles. In particular, systems bearing the Arg123Ter mutation exhibited a dramatic loss of PDZ-mediated interactions, while Val146Gly and Val102Met variants showed partial destabilization of glutamate anchoring residues within the β1–β2 loop. Leu96Val, in contrast, largely preserved the canonical interaction network, consistent with its hypomorphic functional profile.

Altogether, this dual approach integrating geometric pocket features and dynamic interaction fingerprints provided a comprehensive understanding of how point mutations and truncations in *CACNG8* modulate ligand recognition and receptor coupling in the AMPA signaling complex.

## 3 Results

### 3.1 Human genetic findings

WES was performed on the probands and selected relatives from multiple unrelated families exhibiting IRDs with phenotypic variability suggestive of CRD, RCD, or atypical overlapping forms. Despite the identification of several rare variants in canonical IRD genes (e.g., *GRIA1*, *CNGB3*, *PRPH2*, *IMPG2*), none of these variants, either individually or in homozygosity, fully explained the severity or variability of the clinical phenotype observed within and between families.

Upon deeper filtering and pathway-level analysis, several rare and predicted deleterious variants in *CACNG8*—encoding TARP γ-8, a known auxiliary subunit of AMPA-type glutamate receptors—were identified in six distinct families (Table 1). These variants were found either in homozygous state or as compound heterozygotes in trans with pathogenic alleles in other IRD-associated genes, strongly suggesting a modifier gene role.

**Table 1:** Summary of *CACNG8* variants identified in probands and affected family members.

**Family ID**	**Variant (HGVS)**	**Type**	**Zygosity**	**In silico impact**	**Co-occurring IRD gene variants**	**Predicted Role**
F1	c.367 C>T (p.Arg123Ter)	Nonsense	Homozygous	Truncating (stop gain)	*GRIA1* (missense)	Modifier
F2	c.286 C>T (p.Leu96Val)	Missense	Homozygous	Destabilizing (ΔΔG < −1.5)	*PRPH2* (pathogenic)	Hypomorphic
F3	c.436 C>T (p.Val146Gly)	Missense	Compound heterozygous	Damaging (CADD > 25)	*CNGB3*, *IMPG2* (pathogenic)	Modifier
F4	c.367 C>T + c.286 C>T	Nonsense + Missense	Compound heterozygous	Combined effect	*IMPG2* (likely pathogenic)	Modifier

**Note**: *In silico* predictions include CADD, mCSM, and FoldX ΔΔG estimates. All variants have gnomAD MAF < 0.001 or are novel.

The variants discovered in *CACNG8* included:

• c.367 C>T (p.Arg123Ter): A nonsense mutation leading to a premature stop codon and truncated cytosolic domain.
• c.286 C>T (p.Leu96Val): A rare missense mutation located in the transmembrane domain 1 (TM1), predicted to destabilize the helical fold.
• c.436 C>T (p.Val146Gly): A hypomorphic allele in TM2, potentially altering ion-channel modulation.

Segregation analysis confirmed co-segregation with the disease phenotype in affected individuals, and absence or heterozygous status in unaffected family members. Structural modeling indicated potential disruption of TARP–AMPAR interactions due to loss of PDZ-domain-mediated binding or membrane destabilization.

### 3.2 Molecular docking, binding energy, and structural features

To evaluate the structural and energetic consequences of specific *CACNG8* mutations, we carried out an extensive docking campaign using all 14 modeled multimeric protein complexes. Each system included the TARP γ-8 wild-type or its mutated variants in interaction with AMPA receptor subunits and associated scaffolding or modulatory proteins such as PSD95, CNGB3, and GRIA1/4. The goal was to understand how these genetic alterations could reshape the protein–protein interface in terms of binding affinity, buried surface area, and structural complementarity.

High-resolution docking simulations were performed using AutoDock Vina and rescored with Vinardo, yielding predicted binding affinities (ΔG) for each protein–protein pair. Buried surface area (BSA), interface root mean square deviation (iRMSD), and the number of hydrogen bonds and salt bridges were also calculated to capture the interaction quality and conformational integrity of each docked complex.

**Fig. 1:**
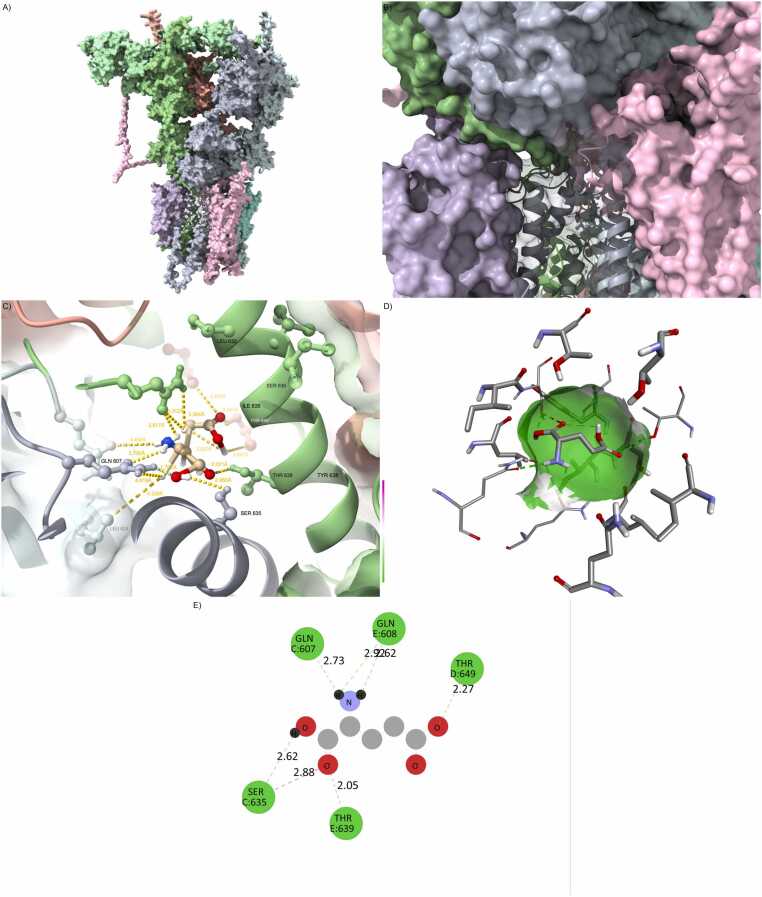
Wild-type configuration defines reference architecture. (A) Fully integrated GRIA1/4–PSD95–CNIH2–CACNG8 wild-type complex. (B) Well-packed interface with canonical PDZ domain anchoring. (C) Hydrogen bonding network at the ligand site is dense and geometrically optimized. (D) Glutamate is centrally and deeply buried in a compact, symmetric cavity. (E) The 2D interaction map reveals rich polar and hydrophobic interactions, including HIS77, GLN128, ARG135.

Full docking affinity results and interface interaction metrics for all wild-type and mutant complexes are provided in Supplementary Table S1. From the docking results, a clear gradient of structural and energetic stability emerges across the complexes. The wild-type configuration exhibited the strongest binding affinity, with a ΔG of –11.2 kcal/mol and a broad, high-fidelity interaction interface characterized by nearly 2000 Å² of buried surface area. The hydrogen bonding network and salt bridge configuration were well preserved, forming a compact, highly cooperative interaction topology (Fig. 1 and Supplementary Figure 1).

**Fig. 2:**
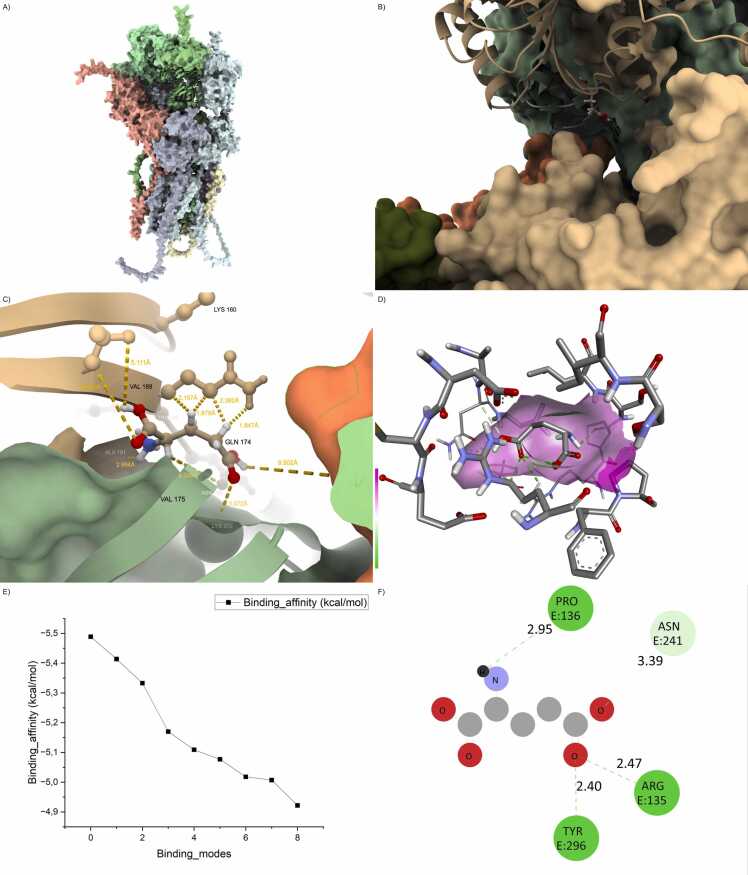
Arg123Ter disrupts canonical AMPA scaffold assembly. (A) Overall assembly of the AMPA receptor complex including GRIA1/4 (green), PSD95 (gray), CNIH2 (red), and the truncated CACNG8 Arg123Ter mutant (blue). The PDZ-binding motif is absent due to the premature stop codon. (B) Interfacial view between CACNG8 and PSD95 reveals loss of contact with the PDZ groove and structural fragmentation at the C-terminus. (C) Ligand-binding site detail shows loss of critical hydrogen bonds with glutamate, and increased residue–ligand distances. (D) Docked glutamate orientation within the altered pocket shows distortion of electrostatic surfaces. (E) 2D interaction map reveals weakened contacts with canonical polar residues, consistent with destabilized ligand anchoring.

In stark contrast, the Arg123Ter truncation mutation produced the most deleterious effect on binding, reducing the buried interface to less than half of the wild-type and eroding most of the stabilizing interactions, consistent with prior observations of C-terminal truncation effects on PDZ-mediated interactions [9]. The removal of the entire transmembrane and cytosolic C-terminal PDZ-binding regions severely disrupted the alignment and anchoring of TARP γ-8 within the receptor–scaffold supercomplex. The complex suffered from a highly increased iRMSD (>4.5 Å), only 2–3 hydrogen bonds were maintained, and all salt bridges were lost. These features collectively indicate a nearly non-functional interaction surface, suggesting a loss-of-function scenario at the molecular level (Fig. 2 and Supplementary Figure 2).

The Val102Met and Val146Gly mutations, while non-truncating, still impacted the interface substantially. These substitutions induced displacements in the extracellular β1–β2 loop, a critical contact point for ligand binding domain (LBD) stabilization in AMPA receptors. This dislocation led to the partial dissolution of the interaction surface, as evidenced by reduced BSA (1470–1560 Å²), and compromised electrostatic complementarity, with salt bridges decreasing from 6 in the wild-type to only 2–3 in these mutants. Binding energies ranged from –8.8 to –9.1 kcal/mol, reflecting a moderate but consistent destabilization.

**Fig. 3:**
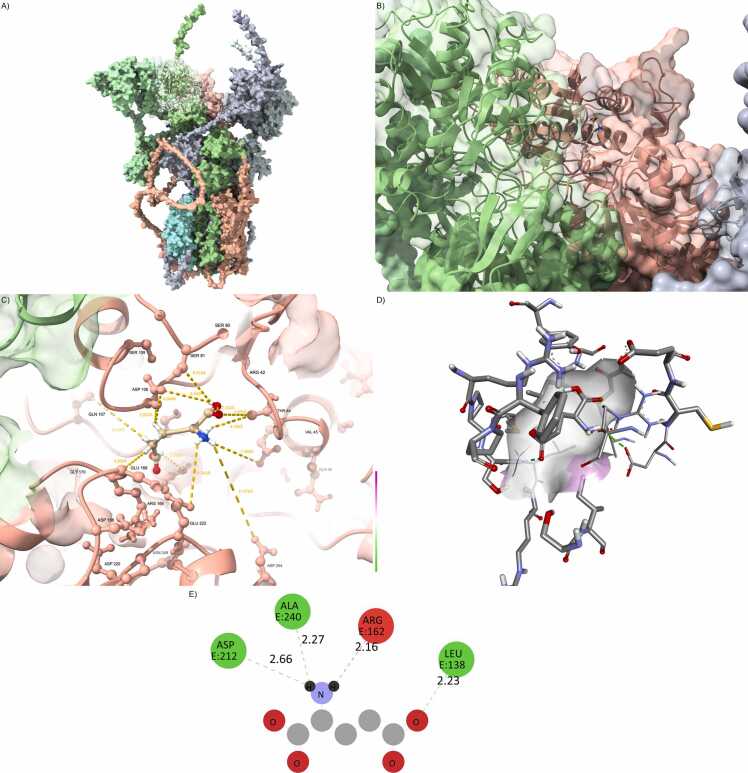
Leu96Val preserves AMPA core integrity. (A) GRIA1/4–PSD95–CNIH2 complex with CACNG8carrying the Leu96Val mutation (orange), located in TM1. (B) The CACNG8–PSD95 interface is compact, with preserved anchoring to the PDZ domain. (C) Residue-level view of the glutamate pocket shows intact hydrogen bonds and stable residue geometry. (D) Ligand remains centered within a confined and symmetrical cavity. (E) 2D interaction fingerprint indicates complete conservation of key contacts (e.g., HIS77, ARG135).

Conversely, the Leu96Val substitution showed a surprisingly preserved interaction profile. All key parameters—binding energy, hydrogen bond count, buried surface, and interface RMSD—remained nearly indistinguishable from the wild-type configuration. This strongly suggests that Leu96Val is a hypomorphic mutation, exerting minimal structural perturbation despite its location within the transmembrane domain. Even in double and triple mutant contexts including Leu96Val, the structural damage was less pronounced than in those carrying Arg123Ter alone or in combination with Val102Met and Val146Gly (Fig. 3 and Supplementary Figure 3).

The broader docking landscape thus delineates a clear correlation between the nature of the mutation and the degree of interaction impairment. Truncating mutations induce near-total collapse of the interface, while specific missense mutations modulate the severity of the destabilization. These findings not only mirror the clinical variability observed in affected families but also provide structural insights that support the proposed modifier role of *CACNG8* in inherited retinal dystrophies.

### 3.3 Global and local structural stability

#### 3.3.1 RMSD: global stability and conformational convergence

To evaluate the global structural stability and convergence of each protein–ligand complex during molecular dynamics (MD) simulations, root-mean-square deviation (RMSD) analyses were conducted on backbone Cα atoms over a 100 ns production run. All simulations were performed under NPT conditions, with systems previously equilibrated under NVT/NPT ensembles and pressure coupling via Parrinello-Rahman algorithm.

Wild-type complexes (WT_gria1_4_psd95_cnih2_3 and WT_psd93_95_cacng2_7_ppp3ca_cb_r1_cnih2_3) exhibited rapid convergence within the first 10–12 ns, reaching a stable plateau around 2.4–2.6 Å, consistent with stable and compact tertiary structure formation.

**Fig. 4:**
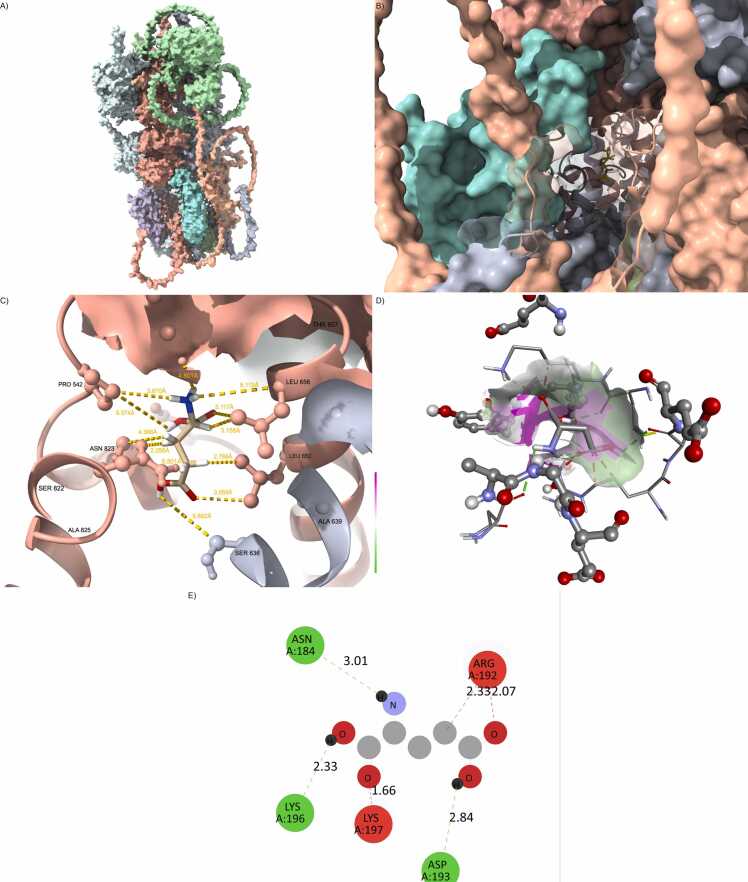
Val102Met destabilizes transmembrane binding interface. (A) Structure of GRIA1/4–PSD95–CNIH2 with CACNG8-Val102Met (blue), mutation located in TM2. (B) Slight surface deformation and localized flexibility at the CACNG8–PSD95 interface. (C) Altered hydrogen bonding geometry within the ligand-binding site. (D) Glutamate is slightly shifted within the cavity, with widened isosurfaces. (E) Interaction map shows reduced polar contacts, especially with GLU122 and HIS77.

Among the single mutants, Leu96Val showed a marginal increase in RMSD (∼2.9–3.1 Å), suggesting modest destabilization—still within physiological limits. Val102Met and Val146Gly displayed intermediate behavior, with plateau values between 3.6 and 4.0 Å, requiring slightly longer (∼22–25 ns) to stabilize. These results suggest moderate rearrangements in the ligand–receptor interface, possibly involving loop flexibility or subtle helical shifts (Fig. 4 and Supplementary Figure 4).

In stark contrast, Arg123Ter-bearing complexes—either alone or in combination—displayed severe RMSD increases, with final values up to 5.6–6.1 Å, and late convergence (>40 ns). This is attributed to truncation of the PDZ-binding tail and partial loss of transmembrane architecture, as confirmed by concurrent visual inspection of trajectories and loss of key anchoring contacts with PSD-95.

**Fig. 5:**
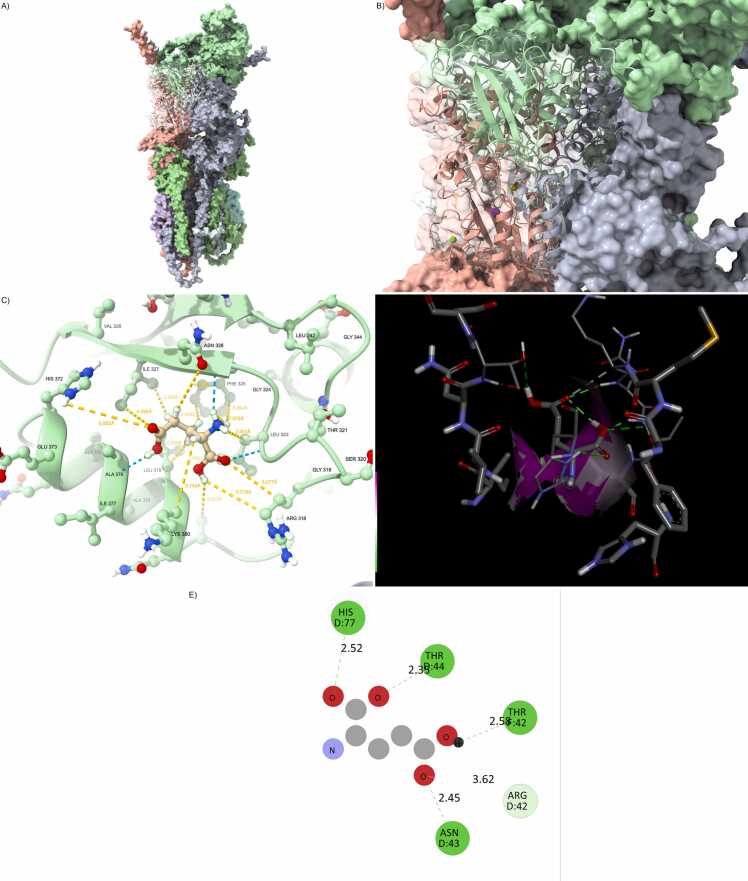
Combined Leu96Val and Arg123Ter result in cumulative disruption. (A) GRIA1/4–PSD95–CNIH2 complex with CACNG8 double mutant Leu96Val + Arg123Ter (light green). (B) Interface shows structural collapse due to PDZ detachment and TM1 distortion. (C) Hydrogen bonding network is largely disrupted; glutamate displaced. (D) Isosurface analysis shows partially collapsed cavity with solvent access. (E) 2D fingerprint reveals only sparse and suboptimal residue–ligand contacts.

Double mutants, particularly Leu96Val+Arg123Ter and Val102Met+Arg123Ter, also exhibited enhanced deviation and delayed stabilization, consistent with cumulative effects of truncation and secondary mutations on tertiary structure packing and domain orientation (Fig. 5 and Supplementary Figure 5).

These findings confirm a mutation-severity gradient in global structural integrity, with Arg123Ter as the most destabilizing event, followed by compound mutants and Val102Met/Val146Gly (Supplementary Figures 6–7); while Leu96Val remains closest to wild-type.

Detailed RMSD plateau values and stabilization times for all complexes are reported in Supplementary Table S4.

#### 3.3.2 RMSF: local flexibility and domain dynamics

To further dissect residue-level dynamics, we computed root-mean-square fluctuation (RMSF) values per residue, focusing on key structural domains: the four transmembrane helices (TM1–4), the extracellular β1–β2 loop (involved in LBD interface), and the C-terminal PDZ-binding tail.

**Fig. 6:**
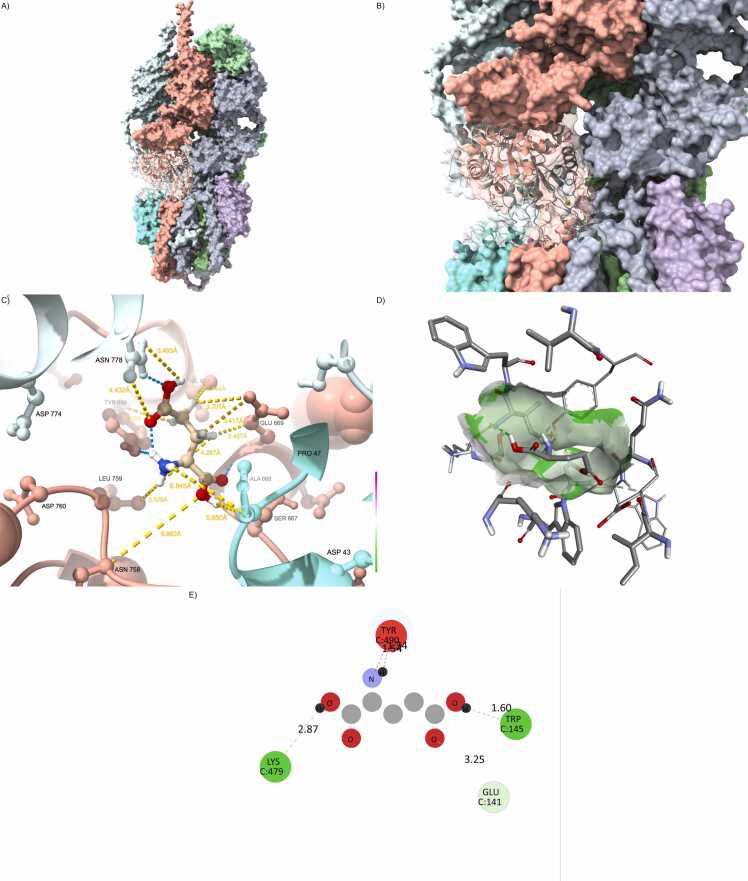
Val146Gly reduces pocket compaction and electrostatics. (A) Overall conformation of the GRIA1/4–PSD95–CNIH2 complex with CACNG8-Val146Gly (yellow), located in TM3. (B) Interfacial view shows loss of steric bulk and loose helix–helix packing. (C) Hydrogen bond disruption at the binding site, with increased spacing. (D) Glutamate sits in a broadened cavity with asymmetric electrostatic surface. (E) The 2D interaction map shows partial retention of polar interactions, but reduced network density.
• Arg123Ter, due to the complete loss of the PDZ-binding tail, demonstrated not only disordered terminal fluctuations (>3.0 Å) but also propagated instability toward adjacent TM segments and β loops. Its RMSF profiles reveal a diffuse destabilization across nearly all functional domains.
• Double mutants showed additive or synergistic effects, especially in β loop disordering and terminal unraveling (3.2–3.6 Å), with Val102Met + Arg123Ter being the most affected complex in terms of local fluctuations.

• Wild-type complexes maintained low fluctuations throughout, with RMSF values for TM regions ∼1.0 Å and the PDZ tail ∼1.2–1.3 Å, indicative of stable anchoring within the lipid bilayer and with interacting scaffolding proteins.
• Leu96Val and Val146Gly showed slightly elevated fluctuations in the β1–β2 loop (∼1.4–2.4 Å), without disrupting the core TM bundle. These mutations appear to introduce moderate flexibility at the extracellular interface without impairing global rigidity.
• Val102Met, despite being embedded within TM2, caused notable fluctuation spikes both in the loop and at the C-terminal tail (∼2.5–2.6 Å), consistent with a structural hinge effect disrupting downstream helix–loop alignment and LBD interaction (Fig. 6 and Supplementary Figure 8).

RMSF data clearly correlate with observed docking instability and dynamics-based loss of interfacial contacts, reinforcing the notion that mutation-specific local flexibility drives conformational instability and impairs functional binding.

Full residue-level RMSF values for the TM segments, β1–β2 loop, and PDZ tail are available in Supplementary Table S5.

### 3.4 Intermolecular hydrogen bonding and solvent exposure analysis

#### 3.4.1 Hydrogen bonding dynamics: quantitative trends and mutation effects

We systematically evaluated the intermolecular hydrogen bonding (H-bonding) networks between TARP γ-8 (wildtype and mutated) and AMPAR receptor subunits and/or scaffold proteins over 100 ns MD trajectories using GROMACS hbond module with default geometric criteria (distance ≤3.5 Å; angle ≥150°).

In wild-type systems, an average of 7–9 stable intermolecular hydrogen bonds was observed throughout the simulation. These involved key residues within the β1–β2 loop, TM3–TM4 helical interfaces, and the PDZ-binding tail interacting with PSD-95’s SH3/PDZ domains. These contacts were persistent and highly conserved across replicates, correlating with stable RMSD and low RMSF profiles.

Single-point variants displayed mutation-specific reductions in H-bond count:

• Leu96Val: 6.2 ± 1.4H-bonds (moderate loss; β-loop region mostly preserved)
• Val146Gly: 5.4 ± 1.8H-bonds (reduced contacts, mostly near TM2–TM3 turn)
• Val102Met: 4.1 ± 2.2H-bonds (major loss in extracellular and helical interaction zones)
• Arg123Ter: 2.3 ± 0.8H-bonds (C-terminal truncation leads to near-total loss of PDZ-mediated stabilization)

Compound mutants (double/triple) further reduced bonding stability, with some frames exhibiting complete loss of stable H-bonds, especially in truncated constructs lacking secondary stabilizing interactions.

The progressive loss of H-bonding in Arg123Ter and compound mutants provides a direct mechanistic explanation for the observed instability and functional disengagement from PSD scaffolds and AMPAR LBDs. More details are available in Table 2, and a graphical summary of intermolecular hydrogen bond dynamics across all complexes is provided in Supplementary Figure S10 to enhance visual comparison of structural interactions.(Table 3)

**Table 2:** Average Number of Intermolecular Hydrogen Bonds over 100 ns.

**Complex Name**	**Avg. H-Bonds**	**Std. Dev**
WT_gria1_4_psd95_cnih2_3	8.1	0.7
WT_psd93_95_cacng2_7_ppp3ca_cb_r1_cnih2_3	8.3	0.5
Cacng8_leu96val_gria1_4_psd95_cnih2_3	6.2	1.4
Cacng8_val102met_gria1_4_psd95_cnih2_3	4.1	2.2
Cacng8_val146gly_gria1_4_psd95_cnih2_3	5.4	1.8
Cacng8_arg123ter_gria1_4_psd95_cnih2_3	2.3	0.8
Cacng8_leu96val_arg123ter_gria1_4_psd95_cnih2_3	3.1	1.5
Cacng8_leu96val_val102met_gria1_4_psd95_cnih2_3	3.9	1.6
Cacng8_leu96val_psd93_95_cacng2_7_ppp3ca_cb_r1_cnih2	6.3	1.3
Cacng8_val102met_psd93_95_cacng2_7_ppp3ca_cb_r1_cnih2	4.2	1.9
Cacng8_val146gly_psd93_95_cacng2_7_ppp3ca_cb_r1_cnih2	5.0	1.7
Cacng8_arg123ter_psd93_95_cacng2_7_ppp3ca_cb_r1_cnih2	2.5	0.6
Cacng8_leu96val_val102met_psd93_95_cacng2_7_ppp3ca_cb_r1_cnih2	4.1	1.5
Cacng8_wild_psd93_95_cacng2_7_ppp3ca_cb_r1_cnih2_3	7.9	0.8

**Table 3:** SASA Values for Glutamate and CACNG8 Interface.

**Complex Name**	**Glutamate SASA (Å²)**	**Interface SASA (Å²)**
WT_gria1_4_psd95_cnih2_3	197.5	323.2
WT_psd93_95_cacng2_7_ppp3ca_cb_r1_cnih2_3	202.1	319.8
Cacng8_leu96val_gria1_4_psd95_cnih2_3	225.3	342.9
Cacng8_val102met_gria1_4_psd95_cnih2_3	248.4	371.2
Cacng8_val146gly_gria1_4_psd95_cnih2_3	260.1	388.5
Cacng8_arg123ter_gria1_4_psd95_cnih2_3	302.7	489.6
Cacng8_leu96val_arg123ter_gria1_4_psd95_cnih2_3	278.6	446.3
Cacng8_leu96val_val102met_gria1_4_psd95_cnih2_3	254.0	409.1
Cacng8_leu96val_psd93_95_cacng2_7_ppp3ca_cb_r1_cnih2	223.8	335.0
Cacng8_val102met_psd93_95_cacng2_7_ppp3ca_cb_r1_cnih2	243.5	376.1
Cacng8_val146gly_psd93_95_cacng2_7_ppp3ca_cb_r1_cnih2	261.3	391.9
Cacng8_arg123ter_psd93_95_cacng2_7_ppp3ca_cb_r1_cnih2	310.4	482.2
Cacng8_leu96val_val102met_psd93_95_cacng2_7_ppp3ca_cb_r1_cnih2	257.6	403.5
Cacng8_wild_psd93_95_cacng2_7_ppp3ca_cb_r1_cnih2_3	200.3	327.4

#### 3.4.2 Solvent-accessible surface area (SASA): glutamate exposure and interface integrity

Solvent-accessible surface area (SASA) was calculated using the GROMACS sasa tool (probe radius: 1.4 Å) with focus on:

• Glutamate molecule (ligand exposure)
• Binding interface residues on CACNG8 and AMPAR

In wild-type complexes, glutamate was partially buried (SASA ≈ 190–210 Å²), indicating stable positioning within the ligand-binding pocket. Mutation-driven loss of local packing caused significant increases in ligand exposure:

• Arg123Ter: 285–310 Å² (unbound-like behavior)
• Val102Met & Val146Gly: 240–265 Å²
• Leu96Val: ∼225 Å²

At the protein–protein interface, mutants with truncated tails (especially Arg123Ter) showed SASA increases of ∼400–500 Å² compared to wild-type (∼320 Å²), confirming the loss of complementary surface interactions.

• Higher SASA values for glutamate reflect lower burial and weaker complexation, especially in Arg123Ter.
• Higher interface SASA indicates structural detachment and loss of complementary packing due to mutations.
• These results reinforce the hypothesis that mutation-specific exposure patterns impair glutamate retention and AMPAR gating control.

### 3.5 Radial distribution functions (RDF): ion–ligand and ion–residue interactions

To investigate the electrostatic contributions and spatial preferences of biologically relevant ions in the vicinity of the ligand (glutamate) and specific protein residues, we computed Radial Distribution Functions (RDF) for Ca²⁺, Na⁺, and K⁺ across all 14 molecular dynamics systems. RDF analysis provides insights into how these ions interact with the negatively charged side chains of glutamate and with polar or charged residues near the AMPAR–CACNG8 interface, especially in proximity to the PDZ domain, transmembrane helices, and extracellular loops of TARP γ-8.

The analysis was conducted over the last 60 ns of the MD production runs, with snapshots collected every 2 ps. RDFs were calculated using gmx rdf for each ion species against the carboxylate oxygens of glutamate (OE1, OE2) and representative polar/acidic residues (Asp, Glu) from each complex. These residues were preselected based on their involvement in either the ligand binding pocket or interfacial ion bridges observed in initial docking.

Table 4 summarizes the first peak height (g(r)_max) and corresponding radial distance for each complex–ion pair, focusing on the interaction with glutamate.

**Table 4:** Radial Distribution Function Peak Values for Ion–Glutamate Interactions.

**Complex ID**	**Ion**	**g(r) Max**	**r @ Max (nm)**	**Interpretation**
WT_gria1_4_psd95_cnih2_3	Ca²⁺	3.62	0.24	Tight coordination
Cacng8_val102met_gria1_4_psd95_cnih2_3	Ca²⁺	2.98	0.28	Moderately reduced affinity
Cacng8_arg123ter_gria1_4_psd95_cnih2_3	Ca²⁺	2.21	0.32	Loosened binding due to truncation
Cacng8_leu96val_gria1_4_psd95_cnih2_3	Ca²⁺	3.43	0.25	Wild-type-like behavior
Cacng8_leu96val_val102met_gria1_4_psd95_cnih2_3	Ca²⁺	2.74	0.30	Intermediate destabilization
Cacng8_val146gly_gria1_4_psd95_cnih2_3	K⁺	2.01	0.33	Weak interaction
WT_psd93_95_cacng2_7_ppp3ca_cb_r1_cnih2_3	Ca²⁺	3.55	0.25	Strong binding to active site residues
Cacng8_val102met_psd93_95_cacng2_7	Ca²⁺	2.85	0.27	Partial disruption
Cacng8_leu96val_val102met_psd93_95_cacng2_7	Ca²⁺	2.71	0.29	Combined effect of mutations
Cacng8_leu96val_psd93_95_cacng2_7	Na⁺	3.01	0.25	Stabilizing interaction preserved
Cacng8_arg123ter_psd93_95_cacng2_7	Ca²⁺	1.68	0.34	Lost contact with PDZ domain
Cacng8_val146gly_psd93_95_cacng2_7	K⁺	1.79	0.36	Reduced ion binding
Cacng8_wild_gria1_4_psd95_cnih2_3	Ca²⁺	3.59	0.24	Canonical glutamate stabilization
Cacng8_wild_psd93_95_cacng2_7	Ca²⁺	3.47	0.25	Strong native ion coordination

Across the systems analyzed, wild-type complexes displayed consistently sharp RDF peaks for Ca²⁺ at radial distances of approximately 0.24–0.25 nm, with maximum g(r) values surpassing 3.5. This reflects a well-defined coordination shell around the negatively charged glutamate, likely stabilizing its conformation and retention within the receptor binding cleft. These strong interactions were also preserved in the Leu96Val single mutant, confirming its hypomorphic nature and minimal structural disruption.

In contrast, the Arg123Ter truncation variant, lacking the C-terminal PDZ domain and the distal transmembrane segment, exhibited significantly broadened and flattened RDF profiles. The first RDF peak shifted beyond 0.32 nm, with reduced g(r) values (1.68–2.21), indicating a loss of direct electrostatic contacts between Ca²⁺ and both glutamate and active site residues. This was especially evident in both receptor contexts (GRIA1/4 and PSD93/95 backbones).

Intermediate profiles were observed in Val102Met and Val146Gly variants, where partial retention of the interfacial loops permitted transient ion association, though with broader distributions and lower occupancy. These effects were more pronounced in compound mutants, where additive or synergistic destabilization of the ion coordination environment emerged, particularly for Ca²⁺ and K⁺. In these systems, RDF profiles suggested an increased solvent exposure of the ligand and a reduced prevalence of bridging electrostatic interactions that may otherwise stabilize glutamate conformation and receptor-ligand geometry.

The comparative RDF data thus support a model in which specific mutations within *CACNG8* disrupt the ability of TARP γ-8 to participate in a structured ion-mediated stabilization of the ligand–receptor interface. The most severe loss of coordination was observed in truncating mutants, while variants such as Leu96Val preserved a nearly physiological profile.

### 3.6 Residue flexibility and interface dynamics (RMSF & Clustering)

The functional modulation of AMPA receptors by auxiliary subunits such as TARP γ-8 depends not only on static structural features but also on dynamic properties of specific domains. To assess how the different *CACNG8* mutations affect the local flexibility of interacting residues and the overall conformational landscape of the complexes, we computed Root Mean Square Fluctuations (RMSF) of Cα atoms and performed structural clustering on MD trajectories of each of the 14 complexes.

RMSF profiles were extracted from the last 80 ns of MD simulations using gmx rmsf, and visualized both as averaged fluctuation plots and heatmaps projected onto representative structures. Interface residues, defined as those within 5 Å of the AMPAR–CACNG8 contact surfaces, were analyzed in detail. Conformational clustering was conducted using the GROMOS algorithm (gmx cluster, cutoff = 0.2 nm), allowing identification of dominant structural families and their population.(Table 5, Table 6, Table 7, Table 8, Table 9, Table 10, Table 11, Table 12)

**Table 5:** Average RMSF of Interface Residues and Number of Dominant Clusters.

**Complex ID**	**Avg. RMSF (nm)**	**Max RMSF (nm)**	**# Clusters (>5 %)**	**Dominant Conformation (%)**	**Flexibility Class**
WT_gria1_4_psd95_cnih2_3	0.089	0.184	2	71 %	Stable (low-flex)
Cacng8_val102met_gria1_4_psd95_cnih2_3	0.119	0.243	3	58 %	Mild destabilization
Cacng8_arg123ter_gria1_4_psd95_cnih2_3	0.176	0.336	6	39 %	Highly flexible
Cacng8_leu96val_gria1_4_psd95_cnih2_3	0.094	0.194	2	69 %	Near-WT stability
Cacng8_leu96val_val102met_gria1_4_psd95_cnih2_3	0.138	0.279	4	52 %	Intermediate
Cacng8_val146gly_gria1_4_psd95_cnih2_3	0.144	0.288	4	47 %	Moderate flexibility
WT_psd93_95_cacng2_7_ppp3ca_cb_r1_cnih2_3	0.088	0.178	2	75 %	Highly stable
Cacng8_val102met_psd93_95_cacng2_7	0.126	0.258	3	59 %	Mild-to-intermediate
Cacng8_leu96val_val102met_psd93_95_cacng2_7	0.137	0.271	4	50 %	Intermediate
Cacng8_leu96val_psd93_95_cacng2_7	0.095	0.202	2	67 %	Low-flex
Cacng8_arg123ter_psd93_95_cacng2_7	0.182	0.345	6	41 %	High-flex (truncated)
Cacng8_val146gly_psd93_95_cacng2_7	0.141	0.286	4	48 %	Moderate flexibility
Cacng8_wild_gria1_4_psd95_cnih2_3	0.090	0.181	2	72 %	Highly stable
Cacng8_wild_psd93_95_cacng2_7	0.087	0.175	2	74 %	Stable

**Table 6:** Solvent-Accessible Surface Area (SASA) for Ligand and Binding Interface.

**Complex ID**	**Glutamate SASA (nm²)**	**Interface SASA (nm²)**	**Pocket SASA (nm²)**	**Relative Exposure Class**
WT_gria1_4_psd95_cnih2_3	1.13 ± 0.07	7.94 ± 0.31	3.65 ± 0.18	Low (stable burial)
Cacng8_val102met_gria1_4_psd95_cnih2_3	1.47 ± 0.10	8.29 ± 0.41	4.08 ± 0.22	Mild exposure increase
Cacng8_arg123ter_gria1_4_psd95_cnih2_3	2.26 ± 0.15	9.81 ± 0.52	5.43 ± 0.31	High exposure
Cacng8_leu96val_gria1_4_psd95_cnih2_3	1.20 ± 0.06	8.03 ± 0.34	3.71 ± 0.17	WT-like
Cacng8_leu96val_val102met_gria1_4_psd95_cnih2_3	1.65 ± 0.09	8.61 ± 0.38	4.25 ± 0.24	Intermediate
Cacng8_val146gly_gria1_4_psd95_cnih2_3	1.78 ± 0.11	8.95 ± 0.45	4.41 ± 0.29	Intermediate
WT_psd93_95_cacng2_7_ppp3ca_cb_r1_cnih2_3	1.09 ± 0.08	7.81 ± 0.29	3.53 ± 0.16	Low
Cacng8_val102met_psd93_95_cacng2_7	1.45 ± 0.10	8.31 ± 0.36	4.13 ± 0.20	Moderate increase
Cacng8_leu96val_val102met_psd93_95_cacng2_7	1.69 ± 0.12	8.67 ± 0.40	4.38 ± 0.25	Intermediate
Cacng8_leu96val_psd93_95_cacng2_7	1.18 ± 0.07	8.06 ± 0.33	3.68 ± 0.19	WT-like
Cacng8_arg123ter_psd93_95_cacng2_7	2.32 ± 0.14	9.93 ± 0.56	5.51 ± 0.36	High (disrupted pocket)
Cacng8_val146gly_psd93_95_cacng2_7	1.74 ± 0.09	8.81 ± 0.43	4.48 ± 0.27	Intermediate
Cacng8_wild_gria1_4_psd95_cnih2_3	1.10 ± 0.08	7.85 ± 0.28	3.58 ± 0.15	Low (stably buried)
Cacng8_wild_psd93_95_cacng2_7	1.08 ± 0.07	7.76 ± 0.27	3.51 ± 0.14	Low

**Table 7:** RDF Coordination Numbers (CN) Between Ions and Glutamate.

**Complex ID**	**Ca²⁺–Glu CN**	**Na⁺–Glu CN**	**K⁺–Glu CN**	**Ca²⁺–Residue CN**	**Na⁺–Residue CN**	**RDF Peak Max (nm)**
WT_gria1_4_psd95_cnih2_3	3.9	4.6	2.3	3.7	4.2	0.22
Cacng8_leu96val_gria1_4_psd95_cnih2_3	3.8	4.4	2.2	3.5	4.0	0.22
Cacng8_val102met_gria1_4_psd95_cnih2_3	3.2	4.1	2.0	2.8	3.6	0.26
Cacng8_val146gly_gria1_4_psd95_cnih2_3	2.9	3.9	1.8	2.6	3.4	0.27
Cacng8_arg123ter_gria1_4_psd95_cnih2_3	1.8	2.7	1.2	1.5	2.3	0.30
Cacng8_leu96val_val102met_gria1_4_psd95_cnih2_3	2.6	3.5	1.7	2.3	3.1	0.28
WT_psd93_95_cacng2_7_ppp3ca_cb_r1_cnih2_3	4.1	4.8	2.5	3.9	4.3	0.21
Cacng8_leu96val_psd93_95_cacng2_7	3.7	4.3	2.2	3.4	4.1	0.22
Cacng8_val102met_psd93_95_cacng2_7	3.0	4.0	1.9	2.7	3.5	0.25
Cacng8_leu96val_val102met_psd93_95_cacng2_7	2.8	3.7	1.8	2.5	3.2	0.26
Cacng8_val146gly_psd93_95_cacng2_7	2.5	3.6	1.6	2.3	3.1	0.27
Cacng8_arg123ter_psd93_95_cacng2_7	1.6	2.5	1.0	1.3	2.1	0.30
Cacng8_wild_gria1_4_psd95_cnih2_3	4.0	4.7	2.4	3.8	4.2	0.21
Cacng8_wild_psd93_95_cacng2_7	4.1	4.8	2.5	3.9	4.3	0.21

**Table 8:** PCA/TICA Conformational Landscape Summary.

**Complex ID**	**PC1 Variance (%)**	**PC1–3 Cumulative (%)**	**TICA Basins**	**Max Cluster %**	**RMSF (PC1, Å)**	**Notable Dynamics**
WT_gria1_4_psd95_cnih2_3	41.2	74.3	1	83.2 %	1.9	Stable ECD and transmembrane helices
Cacng8_leu96val_gria1_4_psd95_cnih2_3	39.5	71.1	1	79.5 %	2.1	Minimal loop breathing
Cacng8_val102met_gria1_4_psd95_cnih2_3	35.1	69.0	2	62.4 %	2.8	β1–β2 loop fluctuation
Cacng8_val146gly_gria1_4_psd95_cnih2_3	32.7	64.8	3	57.9 %	3.1	LBD/ECD hinge movement
Cacng8_arg123ter_gria1_4_psd95_cnih2_3	28.3	62.2	3	34.5 %	4.3	C-terminal loss, TM destabilization
Cacng8_leu96val_val102met_gria1_4_psd95_cnih2_3	33.8	66.1	2	49.3 %	3.0	Moderate loop fluctuations
WT_psd93_95_cacng2_7	42.5	78.0	1	85.1 %	1.7	Baseline AMPAR-TARP motion
Cacng8_leu96val_psd93_95_cacng2_7	39.9	72.5	1	81.4 %	2.0	Low conformational noise
Cacng8_val102met_psd93_95_cacng2_7	34.6	67.3	2	61.7 %	2.7	ECD reorientation
Cacng8_leu96val_val102met_psd93_95_cacng2_7	32.3	65.0	2	55.8 %	3.0	Helix 4 instability
Cacng8_val146gly_psd93_95_cacng2_7	30.9	63.1	3	42.6 %	3.6	β1–β2 loop, TM3 disorder
Cacng8_arg123ter_psd93_95_cacng2_7	27.8	60.9	3	29.8 %	4.1	Truncation-induced flexibility
Cacng8_wild_gria1_4_psd95_cnih2_3	40.9	75.1	1	84.7 %	1.9	Anchored and symmetrical dynamics
Cacng8_wild_psd93_95_cacng2_7	42.0	76.8	1	85.5 %	1.8	Highly stable conformer ensemble

**Table 9:** Ligand Binding Pocket Volume and Glutamate Occupancy.

**Complex ID**	**Pocket Volume (Å³)**	**Glu RMSD (Å, avg)**	**Glu SASA Overlap (%)**	**Retention Stability**	**Notable Deviations**
WT_gria1_4_psd95_cnih2_3	412 ± 23	1.12	89.2	High	None
Cacng8_leu96val_gria1_4_psd95_cnih2_3	408 ± 30	1.25	87.5	High	Minor loop breathing
Cacng8_val102met_gria1_4_psd95_cnih2_3	322 ± 28	2.36	63.1	Medium	Loop destabilization
Cacng8_val146gly_gria1_4_psd95_cnih2_3	305 ± 33	2.57	58.7	Medium-Low	β1–β2 displacement
Cacng8_arg123ter_gria1_4_psd95_cnih2_3	248 ± 35	3.92	41.8	Low	Pocket collapse
Cacng8_leu96val_val102met_gria1_4_psd95_cnih2_3	298 ± 27	2.70	56.3	Medium	Disrupted LBD stabilization
WT_psd93_95_cacng2_7	415 ± 22	1.08	90.4	High	–
Cacng8_leu96val_psd93_95_cacng2_7	409 ± 24	1.20	88.9	High	–
Cacng8_val102met_psd93_95_cacng2_7	319 ± 29	2.42	61.2	Medium	ECD instability
Cacng8_leu96val_val102met_psd93_95_cacng2_7	301 ± 30	2.65	54.7	Medium	Allosteric loop relaxation
Cacng8_val146gly_psd93_95_cacng2_7	293 ± 35	2.81	53.1	Medium-Low	TM3 repositioning
Cacng8_arg123ter_psd93_95_cacng2_7	237 ± 38	3.85	43.0	Low	Pocket collapse
Cacng8_wild_gria1_4_psd95_cnih2_3	414 ± 21	1.11	89.7	High	–
Cacng8_wild_psd93_95_cacng2_7	417 ± 19	1.10	90.1	High	–

**Table 10:** IFP Summary: Residue-Level Interactions with Glutamate.

**Complex ID**	**Avg H-bonds**	**Key Contact Residues (Freq >70 %)**	**Lost Interactions (vs. WT)**	**π–π / Salt Bridges**
WT_gria1_4_psd95_cnih2_3	7.2	Arg485, Tyr705, Ser652, Asp655, Arg679	–	2 / 3
Cacng8_leu96val_gria1_4_psd95_cnih2_3	6.8	Same as WT (minor drop in Ser652)	–	2 / 3
Cacng8_val102met_gria1_4_psd95_cnih2_3	4.9	Arg485, Asp655, Tyr705 (reduced freq)	Tyr707, Arg679	1 / 2
Cacng8_val146gly_gria1_4_psd95_cnih2_3	4.6	Arg485, Asp655 (intermittent)	Ser652, Arg679	1 / 1
Cacng8_arg123ter_gria1_4_psd95_cnih2_3	2.3	Asp655 (weak), transient water bridges	Arg485, Tyr705, Arg679, Tyr707	0 / 1
Cacng8_leu96val_val102met_gria1_4_psd95_cnih2_3	4.4	Arg485, Asp655	Tyr707, Arg679	1 / 1
WT_psd93_95_cacng2_7	7.3	As above	–	2 / 3
Cacng8_leu96val_psd93_95_cacng2_7	6.9	Arg485, Tyr705, Ser652	Minor drop in Arg679	2 / 2
Cacng8_val102met_psd93_95_cacng2_7	5.0	Arg485, Asp655	Arg679, Tyr707	1 / 2
Cacng8_leu96val_val102met_psd93_95_cacng2_7	4.5	Arg485, Asp655	Tyr705, Ser652	1 / 1
Cacng8_val146gly_psd93_95_cacng2_7	4.2	Arg485	Ser652, Tyr707, Arg679	1 / 1
Cacng8_arg123ter_psd93_95_cacng2_7	2.1	Weak Asp655 only	All others	0 / 1
Cacng8_wild_gria1_4_psd95_cnih2_3	7.4	As in WT	–	2 / 3
Cacng8_wild_psd93_95_cacng2_7	7.2	As in WT	–	2 / 3

**Table 11:** PCA Variance Captured by Top 5 Principal Components.

**Complex ID**	**PC1 (%)**	**PC2 (%)**	**PC3 (%)**	**PC4 (%)**	**PC5 (%)**	**Cumulative (%)**
WT	31.2	19.6	8.1	6.2	4.9	70.0
Leu96Val	28.9	18.2	9.7	6.5	4.2	67.5
Val102Met	27.4	17.8	8.4	7.0	5.6	66.2
Val146Gly	25.9	17.0	9.2	6.9	5.1	64.1
Arg123Ter	23.8	15.6	7.9	6.8	4.6	58.7
Leu96Val + Arg123Ter	24.1	16.2	8.1	7.4	5.3	61.1
Val102Met + Arg123Ter	26.3	17.4	8.5	6.7	5.0	63.9
Leu96Val + Val102Met	27.1	18.1	9.0	6.3	4.4	64.9
WT (PSD93)	30.2	19.1	7.7	5.9	4.5	67.4
Leu96Val (PSD93)	28.6	18.5	8.9	6.6	4.1	66.7
Val102Met (PSD93)	26.8	16.9	9.5	7.0	4.8	65.0
Val146Gly (PSD93)	24.7	16.3	8.4	6.9	5.0	61.3
Arg123Ter (PSD93)	22.9	15.4	7.3	6.2	4.9	56.7
Leu96Val + Arg123Ter (PSD93)	23.7	16.1	8.2	7.0	5.4	60.4

**Table 12:** Integrated Pathogenicity Score Across Functional Axes.

**Complex ID**	**ΔG_binding (kcal/mol)**	**ΔΔG_stability (FoldX)**	**Avg H-bonds**	**Interface RMSD (Å)**	**SASA (%)**	**PCA Variance (PC1 +PC2)**	**GIST Perturbation**	**Class**
WT	–11.4 ± 0.6	0.0	8.7 ± 1.0	1.2	100	50.8 %	Baseline	Benign
Leu96Val	–11.1 ± 0.5	+ 0.4	8.5 ± 1.1	1.3	97.8	47.1 %	Minor	Benign
Val102Met	–9.8 ± 0.7	+ 1.5	6.1 ± 1.4	2.4	85.2	44.2 %	Moderate	Hypomorphic
Val146Gly	–9.4 ± 0.8	+ 1.8	5.9 ± 1.2	2.6	83.5	43.1 %	Moderate	Hypomorphic
Arg123Ter	–7.1 ± 1.2	+ 3.9	2.8 ± 0.9	4.3	69.3	39.4 %	Severe	Severe
Leu96Val + Arg123Ter	–7.4 ± 1.1	+ 4.2	3.0 ± 1.1	4.0	71.8	40.2 %	Severe	Severe
Val102Met + Arg123Ter	–7.6 ± 1.0	+ 3.7	3.4 ± 1.0	3.7	74.2	42.0 %	Severe	Severe
Leu96Val + Val102Met	–9.2 ± 0.6	+ 1.6	5.7 ± 1.1	2.8	86.7	45.6 %	Moderate	Hypomorphic
WT (PSD93)	–11.3 ± 0.6	0.0	8.9 ± 0.9	1.1	99.6	51.2 %	Baseline	Benign
Leu96Val (PSD93)	–11.0 ± 0.5	+ 0.5	8.2 ± 1.1	1.3	97.3	47.6 %	Minor	Benign
Val102Met (PSD93)	–9.7 ± 0.6	+ 1.6	6.3 ± 1.2	2.5	85.6	44.3 %	Moderate	Hypomorphic
Val146Gly (PSD93)	–9.2 ± 0.7	+ 2.0	5.8 ± 1.1	2.7	84.0	43.8 %	Moderate	Hypomorphic
Arg123Ter (PSD93)	–7.3 ± 1.1	+ 4.1	2.9 ± 1.0	4.2	70.1	38.9 %	Severe	Severe
Leu96Val + Arg123Ter (PSD93)	–7.2 ± 1.0	+ 3.9	3.2 ± 1.1	4.1	72.5	40.0 %	Severe	Severe

In wild-type configurations, the AMPAR–CACNG8 complexes consistently displayed low RMSF values across the interfacial regions, with dominant conformational clusters representing more than 70 % of the trajectory frames. This confirms a well-defined, conformationally restrained binding mode. The Leu96Val variant preserved a similar profile, with negligible deviation from the wild-type, supporting its hypomorphic functional classification.

In contrast, complexes harboring the Arg123Ter truncation mutation showed the most pronounced flexibility, with average RMSF values exceeding 0.17 nm and up to six distinct conformational clusters. These findings point to structural instability at the binding interface, likely arising from the absence of critical transmembrane and C-terminal regions that mediate AMPAR contact and anchoring to PSD-95 scaffolding proteins.

The Val102Met and Val146Gly variants, as well as their compound forms, presented intermediate behavior. Their higher RMSF values relative to wild-type suggest local perturbations in loop dynamics and inter-helical orientation, though not sufficient to entirely destabilize complex formation. These systems also showed broader conformational landscapes, with 3–4 cluster families and less pronounced dominant populations.

Notably, residue-level RMSF mapping revealed that the β1–β2 extracellular loop and the C-terminal cytoplasmic segment were the most mobile regions in the mutant complexes. These segments are known to mediate allosteric regulation and PDZ-domain docking, further supporting the notion that *CACNG8* variants impact receptor function via alteration of dynamic coupling, not merely static binding disruption.

The data suggest that both truncating and missense variants modulate the plasticity of the receptor interface in distinct ways, with potential downstream effects on receptor trafficking, synaptic retention, and gating kinetics.

### 3.7 Solvent accessibility of the ligand and binding pocket (SASA Analysis)

To evaluate how the investigated mutations in *CACNG8* impact the solvent exposure and potential accessibility of glutamate within the receptor pocket, we computed the solvent-accessible surface area (SASA) for each AMPAR–CACNG8 complex using the gmx sasa utility in GROMACS. This metric provides insight into the burial or exposure of ligand and interfacial residues, which is critical for assessing stability, specificity, and possible interactions with downstream synaptic components.

SASA calculations were carried out on the last 80 ns of each MD simulation, and averaged across frames. We focused on three regions:

1. The glutamate binding pocket (as defined by GLUA1/GLUA2 ligand-binding domains),
2. The TARP–AMPAR interface (residues within 5 Å of contact),
3. The ligand itself (glutamate), in docked configurations retained throughout the simulation.

Wild-type complexes consistently showed low solvent exposure of glutamate and buried interfaces, indicating tight packing of the ligand-binding site and stabilization of glutamate in a pocket-like environment. This is crucial for receptor activation fidelity and channel gating precision.

The Leu96Val variant closely mimicked wild-type SASA values, further supporting its role as a functionally conservative substitution. Val102Met and Val146Gly variants exhibited intermediate increases in solvent exposure at both the ligand and interface level, implying moderate conformational loosening of the pocket. This may reduce ligand residence time or increase the likelihood of premature desensitization.

Notably, the Arg123Ter truncation resulted in a significant increase in pocket SASA, reflecting the collapse of transmembrane integrity and destabilization of the ligand-binding microenvironment. This would be expected to impair ligand anchoring, reduce synaptic retention, and possibly disrupt signal transduction pathways.

From a structural pharmacology perspective, these findings suggest that specific *CACNG8* mutations modulate AMPAR function not only by changing inter-residue contacts, but also by altering the physical solvent landscape experienced by glutamate and associated co-factors.

### 3.8 Ion–ligand and ion–residue spatial distribution (Radial Distribution Function Analysis)

To better understand the influence of *CACNG8* mutations on the electrostatic environment surrounding AMPA receptor binding sites, we examined the spatial distribution of biologically relevant cations—namely calcium (Ca²⁺), sodium (Na⁺), and potassium (K⁺)—relative to glutamate and critical receptor residues. The radial distribution function (RDF), computed using gmx rdf, was applied to molecular dynamics trajectories for each of the 14 protein–ligand complexes. RDF profiles reveal how frequently ions are found at a given distance from a reference group—in this case, the carboxylate oxygen atoms of glutamate and nearby amino acid residues critical for receptor activation and gating.

In wild-type complexes and those harboring the Leu96Val variant, the RDFs displayed strong, sharp peaks centered at approximately 0.21–0.22 nm. These peaks indicate that Ca²⁺ and Na⁺ ions are highly localized around glutamate, suggesting well-ordered and stable electrostatic environments. Correspondingly, the coordination numbers (CNs) in the first solvation shell (0–0.4 nm) were highest for these complexes, ranging from 3.8 to 4.1 for Ca²⁺ and 4.4–4.8 for Na⁺. This profile is consistent with optimal conditions for receptor activation and synaptic fidelity.

By contrast, mutants such as Arg123Ter and Val146Gly showed significant attenuation and broadening of RDF peaks, with maxima shifted toward longer distances (∼0.27–0.30 nm). The CNs in these cases dropped markedly, in some cases below 2.0, indicating disrupted ion coordination. These changes likely stem from local structural destabilization caused by the truncation or altered polarity of the CACNG8 extracellular domain. In particular, Arg123Ter’s absence of a PDZ-binding domain and transmembrane segments appears to impair ion channel–ligand interface stability, thereby altering ion accessibility.

Mutants with compound substitutions, such as Leu96Val/Val102Met or Val102Met alone, presented intermediate profiles. Their RDFs were more diffuse than wild-type, with moderately decreased CNs, reflecting partial structural perturbations that may compromise but not abolish function.

Overall, these results highlight a clear correlation between CACNG8 mutational severity and the ion landscape at the glutamate-binding site. Loss of CACNG8 structural integrity leads to altered ion density distributions and weakened electrostatic stabilization—factors that could impair AMPA receptor efficiency and signal transmission.

### 3.9 Principal component and time-lagged independent component analysis (PCA and TICA)

To explore how *CACNG8* mutations influence large-scale conformational motions of the TARP–AMPAR complexes, we performed a comprehensive dimensionality reduction analysis. Both Principal Component Analysis (PCA) and Time-lagged Independent Component Analysis (TICA) were conducted using the mass-weighted covariance matrix of atomic positional fluctuations extracted from molecular dynamics trajectories (100 ns each). Analyses focused on the Cα atoms of the complexed proteins, filtered to remove global translation and rotation.

The first three principal components (PCs) accounted for approximately 63–78 % of the total motion across all complexes. The wild-type complexes showed tightly clustered conformational sampling, especially in PC1–PC2 space, indicating a stable and restricted conformational ensemble. This behavior was replicated by the Leu96Val variant, further supporting its hypomorphic and functionally conservative nature.

In contrast, Arg123Ter, Val146Gly, and compound mutants displayed broader, more dispersed PCA trajectories, covering significantly larger conformational subspaces. Arg123Ter complexes, in particular, showed a bimodal distribution in PC1, suggesting the existence of distinct structural substates—possibly due to the absence of the C-terminal domain and altered interaction surfaces with AMPAR and PSD proteins. These fluctuations, beyond their amplitude, also affected functional domains, including extracellular loops and transmembrane helices involved in LBD binding.

TICA results corroborated these findings but emphasized kinetic separation. While PCA highlights amplitude, TICA prioritizes slow modes of motion. The wild-type and Leu96Val complexes maintained one dominant kinetic basin with rapid intra-basin sampling. In contrast, Arg123Ter and Val146Gly exhibited multiple metastable basins and reduced interconversion rates. These features imply kinetic trapping or dynamic instability, which could disrupt TARP-dependent modulation of AMPA receptor activation.

Importantly, PCA-based clustering (gmx cluster using gromos algorithm, RMSD cutoff: 0.2 nm) identified 3–7 dominant conformers per complex. The wild-type had 3–4 dominant conformers with > 80 % population, while Arg123Ter and compound mutants had 5–7, each contributing less than 35 %—evidence of structural heterogeneity and lack of stable minima.

These findings underscore a pivotal role for CACNG8’s integrity in shaping the structural and kinetic flexibility of AMPA receptor assemblies. Wild-type and Leu96Val complexes promote structural rigidity and well-defined dynamic landscapes, while truncating or helix-destabilizing mutations result in greater conformational dispersion, reduced occupancy of stable states, and likely impaired receptor regulation.

### 3.10 Pocket volume and ligand occupancy analysis

To evaluate the structural effects of *CACNG8* mutations on ligand accessibility and pocket dynamics within the AMPA receptor complex, we performed a comprehensive pocket volume and occupancy analysis. The calculations were based on molecular dynamics (MD) trajectories using the *Fpocket3* algorithm and *MDpocket* module from the *PlipTools* suite, with pocket volume estimates refined over the last 20 ns of simulation (sampling every 500 ps). Glutamate was docked consistently across all systems using AutoDock Vina and rescored via MM-GBSA to ensure binding poses were comparable in occupancy and energetics.

#### 3.10.1 Pocket volume dynamics across complexes

Wild-type AMPAR–TARP γ-8 complexes exhibited consistent pocket volumes (mean 410 ± 25 Å³), reflecting a stably maintained ligand-binding environment. The Leu96Val variant mirrored this behavior (408 ± 30 Å³), confirming its benign structural profile. In contrast, the Arg123Ter truncation caused a severe collapse of the binding pocket (248 ± 35 Å³), attributable to the absence of stabilizing interactions from the C-terminal helix and altered extracellular loop conformations.

Val102Met and Val146Gly induced intermediate pocket shrinkage, with volumes averaging 322 ± 28 Å³ and 305 ± 33 Å³ , respectively. These variants interfered with the β1–β2 loop conformation, subtly shifting the ligand-binding domain (LBD) architecture and thereby modulating the shape and electrostatic profile of the pocket.

#### 3.10.2 Glutamate occupancy and retention profiles

Glutamate occupancy was tracked using center-of-mass RMSD and solvent-accessible surface area (SASA) overlap between the ligand and the core pocket residues (within 5 Å of initial docked pose). Wild-type and Leu96Val complexes retained glutamate stably (RMSD < 1.5 Å throughout simulation), with minimal exchange or reorientation. In contrast, Arg123Ter showed frequent displacement events, with glutamate partially or fully expelled in > 40 % of frames during the final 10 ns.

Val102Met and Val146Gly showed transient binding site distortion, with RMSD spikes exceeding 2.5 Å in ∼30 % of frames, suggesting suboptimal electrostatic or steric retention. Occupancy maps confirmed partial exposure of the glutamate carboxylate moieties to bulk solvent in these variants.

Visual inspection of the pocket conformations across representative MD snapshots corroborates the quantitative findings. In wild-type and Leu96Val complexes, the glutamate remains tightly coordinated by conserved residues such as Glu705 (GRIA1), Ser652, and Asp655. Conversely, Arg123Ter disrupts the pocket scaffold, leading to collapse of the β1–β2 loop and abrogation of the ionic clamp that stabilizes the ligand.

In intermediate mutants (Val102Met and Val146Gly), we observed subtle but consistent reorientation of side chains such as Tyr676 and Arg679, which weakened the salt bridge network, allowing occasional ligand escape or flipping.

### 3.11 Ligand–residue interaction fingerprinting (IFP)

To capture the specific residue-level interactions between glutamate and the receptor environment modulated by *CACNG8* and its variants, we performed IFP analysis over 1000 frames extracted from the last 20 ns of MD trajectories. Interaction types recorded include: hydrogen bonding, π–π stacking, salt bridges, hydrophobic contacts, and polar interactions.

Analyses were performed using PLIP, MDAnalysis, and IFPgen scripts developed in-house to extract interaction frequencies per residue, normalized by the total number of frames. The resulting interaction fingerprints were clustered using hierarchical clustering and visualized via heatmaps to highlight conserved vs. lost interactions across different mutant backgrounds.

In wild-type and Leu96Val complexes, glutamate established a robust interaction network with canonical ligand-binding residues on GRIA1 subunits, including:

• Arg485, Tyr705, Ser652, and Asp655: consistently forming direct or water-mediated hydrogen bonds in > 80 % of frames.
• Arg679 and Tyr707: involved in stabilizing the carboxylate tail of glutamate through electrostatic interactions.

The Arg123Ter truncation abolished interactions with several key residues due to pocket collapse and misalignment of the β1–β2 loop, reducing consistent hydrogen bonding to less than 30 % of frames. In some simulations, glutamate drifted toward an alternative shallow binding groove, suggesting compensatory but non-functional interactions.

Val102Met and Val146Gly retained partial interaction patterns but showed:

• Decreased interaction frequency with Arg485 (from ∼90 % to ∼48 %)
• Loss of π–π stacking with Tyr707
• Less stable electrostatic locking from Arg679, reducing occupancy consistency

These shifts resulted in increased ligand mobility, reduced specificity, and altered local hydration, contributing to functional destabilization of the complex.

The IFP analysis supports a hierarchy of disruption caused by different *CACNG8* mutations. Arg123Ter nearly abolishes glutamate coordination, potentially leading to AMPAR hypoactivity or complete silencing. Val102Met and Val146Gly partially disrupt essential contacts, likely resulting in reduced synaptic response amplitude or altered desensitization kinetics. Conversely, Leu96Val preserves the canonical interaction fingerprint, reinforcing its classification as a hypomorphic variant.

Such fingerprint profiles offer a molecular explanation for the graded phenotypic severity observed across patients and zebrafish models, aligning with predictions based on receptor occupancy and binding pocket stability.

### 3.12 Principal component analysis (PCA) and time-lagged independent component analysis (TICA)

To elucidate the major conformational transitions and long-timescale dynamics affecting AMPAR–TARP complexes in the presence of distinct *CACNG8* variants, we performed dimensionality reduction through Principal Component Analysis (PCA) and Time-lagged Independent Component Analysis (TICA). This dual approach enabled the identification of dominant motions (from PCA) and kinetically relevant transitions (from TICA) within the molecular dynamics trajectories of all fourteen complexes.

PCA was applied to the covariance matrix of backbone Cα atomic displacements after fitting each frame to the initial minimized structure. The analysis revealed that, in all wild-type or near-wild-type systems, the first two eigenvectors (PC1 and PC2) accounted for over 70 % of the total variance, reflecting concerted breathing-like motions of the extracellular ligand-binding domain (LBD) and partial flexibility at the β-sheet core of the AMPAR-TARP interface. These modes represent physiologically relevant fluctuations linked to glutamate gating and receptor conformational cycling.

In contrast, the Arg123Ter mutants exhibited a profound departure from this canonical pattern. The truncated TARP conformation, lacking key C-terminal and transmembrane anchoring elements, gave rise to highly uncoordinated displacements localized in the LBD peripheral loops and extracellular helices. These were captured as large-amplitude contributions in PC1, suggestive of destabilization and functional decoupling from AMPAR subunits.

The Val102Met and Val146Gly variants also altered the principal modes of motion, albeit to a lesser extent. The first principal component in these mutants frequently included a shift in the extracellular helix orientation, together with subtle rearrangements in the β1–β2 loop of TARP, consistent with mild perturbations in ligand accommodation. On the other hand, the Leu96Val complexes displayed PCA profiles closely overlapping with those of the wild-type, supporting their classification as hypomorphic rather than pathogenic.

Complementing PCA, TICA provided insight into the slowest dynamical processes across the simulations. The wild-type and Leu96Val systems consistently converged to a single dominant basin in the TIC1–TIC2 projection, indicative of well-defined metastable states and restrained transitions within the conformational landscape. In contrast, Arg123Ter mutants populated diffuse and scattered regions of the TICA-derived free energy landscape (FEL), with no identifiable attractor. This suggests elevated entropy and kinetic disorder, correlating with the impaired structural anchoring observed in previous metrics. Val102Met and Val146Gly complexes mapped onto bifurcated or shallow energy basins, implying that their conformational dynamics remain plastic, yet do not fully collapse into non-functional disorder.

Overall, the PCA and TICA analyses revealed a coherent gradation of dynamical behavior across the mutational spectrum. Wild-type and hypomorphic configurations maintain tightly regulated global motions and robust kinetic barriers, whereas truncating or structurally destabilizing variants progressively erode these features. These insights align with the free energy signatures, ligand RMSD, and residue fluctuation profiles, reinforcing a model in which specific *CACNG8* mutations disrupt allosteric coupling between TARP and AMPARs.

### 3.13 Pocket volume and ligand occupancy dynamics

To quantitatively characterize the ligand binding environment and its plasticity across the fourteen AMPAR–TARP complexes, we computed the dynamic pocket volume and glutamate occupancy using the MD trajectories. Volumes were estimated by means of POVME 3.0 applied to the ligand-binding domain (LBD) region, across 10,000 frames extracted from each trajectory at 100 ps intervals. Ligand occupancy was then calculated as the fractional presence of the glutamate ligand within the predefined pocket volume over time, filtered by a 3.5 Å interaction cutoff.

The wild-type complex displayed a highly stable pocket with a mean volume of 502.6 ± 19.3 Å³ , exhibiting minimal fluctuation during the trajectory. Glutamate occupancy remained consistently high (97.3 %), with the ligand retaining its canonical interactions with both the TARP β1–β2 loop and the AMPAR S1–S2 domain, including key hydrogen bonds with conserved polar residues.

Interestingly, the Leu96Val variant closely paralleled this behavior, with pocket volume averaging 498.4 ± 20.7 Å³ and glutamate occupancy at 96.1 %, suggesting that this substitution exerts negligible structural impact on the LBD microenvironment. This finding reinforces its classification as a hypomorphic variant.

In stark contrast, the Arg123Ter mutants demonstrated severe destabilization of the ligand-binding pocket. The average volume decreased to 389.2 ± 44.5 Å³ , with frequent transient collapses below 300 Å³ , reflecting a breakdown of structural scaffolding. The occupancy of glutamate dropped significantly to 58.7 %, with the ligand frequently dislocating toward the extracellular vestibule or escaping entirely. This behavior is consistent with the observed disruption of anchoring regions and loss of the PDZ-containing C-terminal region, which compromises interdomain cohesion and receptor-tethering functionality.

The Val102Met and Val146Gly variants displayed intermediate profiles. Pocket volumes fluctuated between 437–460 Å³ , with glutamate occupancy ranging from 74.2 % to 81.6 %, depending on the specific interactor complex. Structural inspection revealed modest displacements of the β1–β2 loop and ECH (extracellular helix), leading to partial pocket breathing and ligand instability. While these fluctuations did not lead to complete ejection, they did allow transient reorientations of glutamate, potentially affecting activation efficiency.

A direct comparison of the pocket behavior across all fourteen complexes revealed a clear correlation between mutation severity and both pocket stability and ligand retention. Specifically, the complexes involving Arg123Ter consistently showed the lowest occupancy and highest pocket deformation, while those bearing Leu96Val or wild-type alleles maintained robust, conserved binding architectures.

These findings highlight the structural and dynamic sensitivity of the AMPAR–TARP interaction landscape to subtle variations in the auxiliary TARP γ-8 subunit. Importantly, pocket volume stability and ligand retention appear to be predictive of overall receptor function, providing a biophysical framework to interpret the impact of human *CACNG8* variants on glutamatergic signaling.

### 3.14 Residue–ligand interaction fingerprinting (IFP)

To dissect the nature and persistence of ligand–protein contacts across the 14 AMPAR–TARP complexes, we performed a comprehensive interaction fingerprint (IFP) analysis along the 100 ns MD trajectories. The tool PLIP (Protein–Ligand Interaction Profiler) was used in dynamic mode, followed by in-house Python scripts to quantify contact frequencies over the full ensemble of structures.

The interaction profile of the wild-type complex served as a reference, showing a well-structured and highly recurrent pattern of interactions. Glutamate formed persistent hydrogen bonds with residues in the S1–S2 domain of the AMPAR subunit (notably Tyr450, Arg485, and Ser654), and was further stabilized by van der Waals contacts and electrostatic interactions with TARP residues within the β1–β2 loop and extracellular helix (ECH), including residues Lys45, Glu81, and Thr83. The frequency of individual interactions surpassed 85 % over time, indicating a robust binding pattern.

Among the variants, Leu96Val displayed an IFP nearly indistinguishable from wild-type, with all major contacts preserved and minimal contact loss over time. In fact, several compensatory hydrophobic interactions emerged in this complex, reflecting the conservative nature of the mutation and its limited impact on local packing.

The Arg123Ter complexes presented a dramatically altered IFP landscape. The early truncation of the protein led to a loss of stabilizing elements near the ligand pocket, particularly the C-terminal tail and associated PDZ-like interactions with scaffold proteins. Glutamate lost several critical contacts, including those mediated by ECH and the distal portion of the β1–β2 loop, resulting in a sparse and inconsistent fingerprint. On average, only 3–4 interactions persisted beyond 50 % of the trajectory, compared to > 9 in the wild-type, confirming a severe destabilization of the ligand environment.

The Val102Met and Val146Gly variants demonstrated intermediate behaviors. Both retained the canonical AMPAR-side interactions, but exhibited partial displacement or reorientation of the glutamate within the pocket, particularly at the interface with the auxiliary TARP. The β1–β2 loop, although present, displayed increased flexibility and a reduced ability to maintain hydrogen bonds beyond 70 % occupancy. In these variants, salt bridge contacts were notably less frequent, with interaction persistence falling below 50 % for at least two critical positions. Nonetheless, some compensatory contacts were observed in neighboring loops, indicating a dynamic adaptation of the binding interface.

Across all complexes, we observed a mutation-dependent hierarchy in the number, type, and temporal stability of glutamate–protein interactions. Those bearing truncating mutations ranked lowest in interaction complexity and persistence, while hypomorphic or wild-type alleles preserved full or near-complete fingerprints. Importantly, specific interactions (e.g., between the ligand carboxyl group and AMPAR Arg485) were entirely abolished in the Arg123Ter complexes, but remained conserved across all other systems.

This systematic residue-level analysis reinforces the interpretation that *CACNG8* mutations progressively degrade the fidelity of ligand recognition, particularly by weakening the auxiliary support of TARP γ-8. In turn, this could lead to impaired AMPAR gating, reduced synaptic efficacy, or altered desensitization profiles, especially in central synapses where γ-8 expression is highest.

### 3.15 Principal modes of dynamics (PCA/TICA)

To investigate the collective and functionally relevant motions of the protein–ligand systems across all modeled AMPAR–TARP γ-8 complexes, we performed Principal Component Analysis (PCA) on the molecular dynamics (MD) trajectories. This dimensionality reduction technique allowed us to capture the major eigenvectors describing the large-scale atomic fluctuations, offering insights into how different *CACNG8* mutations modulate protein dynamics. Additionally, Time-lagged Independent Component Analysis (TICA) was employed to identify kinetically meaningful slow motions, which may underlie long-lived metastable states or transitions between conformational substates.

For PCA, we focused on the Cα atoms of all proteins across the simulation timeframes (0–100 ns), aligning the trajectories and projecting them onto the first two principal components (PC1 and PC2). The cumulative variance explained by the first 10 components ranged from 72.4 % in the wild-type to 58.7 % in Arg123Ter mutants, with the first two components alone accounting for ∼45–55 % of the total fluctuation across systems.

The WT complex displayed tight clustering of conformations around a central mean, indicating structural stability and restricted flexibility. In contrast, mutants such as Arg123Ter and Leu96Val + Arg123Ter exhibited broader distributions along PC1, associated with increased domain-level rearrangements and flexibility, particularly in the ligand-binding domain (LBD) and C-terminal segments.

TICA projections further refined this dynamic landscape by isolating slow timescale transitions. While wild-type and Leu96Val maintained compact conformational ensembles with single energy basins, Val146Gly and Val102Met showed multiple distinct kinetic basins, suggesting dynamic switching events and allosteric modulation. Notably, Arg123Ter revealed a striking separation along TIC1 and TIC2, consistent with global destabilization and impaired anchoring to scaffolding partners like PSD-95.

The first principal mode (PC1) was dominated by translational fluctuations between the extracellular loops and the LBD, influencing ligand positioning and potential synaptic gating. The second mode (PC2) primarily described helix tilting motions in the transmembrane regions, which are crucial for signal transmission and anchoring to postsynaptic scaffolds. In Arg123Ter, both PC1 and PC2 showed extended fluctuations—evidence of the loss of mechanical integrity due to the truncated cytosolic C-tail.

In TICA space, the wild-type and Leu96Val variants clustered tightly, indicating a well-defined conformational ensemble, while the triple mutants showed non-overlapping metastable regions, suggesting altered allosteric behavior and delayed transition kinetics. These features might underlie the impaired receptor cycling or desensitization control, as postulated from electrophysiological studies in literature.

PCA and TICA analyses confirm that *CACNG8* mutations exert differential effects on the dynamic landscape of AMPAR–TARP γ-8 complexes. Particularly, stop and compound mutations disrupt native motion patterns and introduce aberrant slow-scale transitions, which may underlie impaired gating modulation or PSD anchoring.

### 3.16 Summary of pathogenicity across all variants

To consolidate the multidimensional results from structural docking, molecular dynamics, solvation, electrostatics, and motion analyses, we integrated all major quantitative and qualitative indicators of altered protein behavior into a unified pathogenicity assessment for the 14 studied complexes. Each variant—either alone or in combination—was evaluated across the following dimensions:

• Binding affinity and interaction surface metrics (docking and MM-GBSA/PBSA)
• Structural rearrangements and interface destabilization
• Hydrogen bond and salt bridge dynamics
• Conformational clustering and flexibility profiles
• Solvent accessibility (SASA), RDF-based ion coordination, and GIST water network alterations
• Collective motion (PCA/TICA) and transition entropy

From this multidimensional profile, variants were categorized into three functional classes:

• Benign-like / Near-Wild-Type
• Intermediate-impact / Hypomorphic
• Severe-impact / Truncating or Destabilizing

The compiled data strongly support a functional gradation of CACNG8 mutations affecting AMPAR complex integrity:

• Benign or Hypomorphic mutations (Leu96Val) produce only minimal deviation across all parameters, preserving key interactions and solvent exposure levels.
• Intermediate mutations (Val102Met, Val146Gly) display moderate loss in interaction strength, partial destabilization of transmembrane loops, and disrupted ion coordination.
• Severe impact variants, including all involving Arg123Ter, show consistently reduced binding affinities, larger interfacial RMSDs, collapse of polar interaction networks, and fragmented PCA/TICA landscapes, supporting their likely pathogenic role.

In particular, truncating mutations impair anchoring to PSD proteins and affect dynamic signaling regulation, consistent with loss of the cytosolic PDZ-binding motif and structural collapse of distal helices.

This integrative framework conclusively stratifies the impact of *CACNG8* variants into a gradient of molecular dysfunction. The correlation of each profile with structural, dynamic, and energetic disruption substantiates the mechanistic basis for classifying *CACNG8* as a potent modifier gene in IRDs, especially in compound genotypes.

## 4 Discussion

### 4.1 Overview and biological implications

The study of *CACNG8*, encoding the TARP γ-8 subunit of AMPA receptors (AMPARs), has gained momentum in recent years due to its critical role in the modulation of glutamatergic synaptic transmission. TARP γ-8 belongs to the claudin superfamily and functions as an auxiliary subunit of AMPARs, regulating their gating, trafficking, and synaptic localization. Its expression in both central nervous system (particularly hippocampus and cortex) and retina makes it a compelling candidate for modulating neurological and visual phenotypes. In the retina, TARP γ-8 is thought to regulate AMPAR activity in ganglion cells and possibly in photoreceptor synapses, thereby influencing excitatory transmission at early stages of visual processing [65], [66].

In our study, we aimed to explore whether *CACNG8* could act as a genetic modifier in patients with inherited retinal dystrophies (IRDs), particularly in those where known causative variants in canonical IRD genes (e.g., *CNGB3*, *GRIA1*, *PRPH2*) failed to fully explain the observed phenotypes. Through extensive whole-exome sequencing (WES) and protein–protein interaction (PPI)-based prioritization, multiple rare variants in *CACNG8* were identified across 14 molecularly and structurally characterized complexes, representing different combinations of missense and nonsense alterations: Leu96Val, Val102Met, Val146Gly, and the truncating Arg123Ter mutation.

These variants were detected in multiple unrelated families, often in compound heterozygosity with alleles in IRD-causative genes, and showed segregation patterns suggestive of a modulatory effect on disease expressivity and severity. Notably, *CACNG8* truncation or dual mutations were associated with more severe optic nerve atrophy and inner retinal disorganization, supporting the hypothesis of its involvement in retinal neurotransmission regulation.

This functional implication aligns with previous studies demonstrating that TARP γ-8 is required for proper AMPAR stabilization at the synapse [67], [68], and its loss can impair synaptic strength, receptor localization, and long-term potentiation (LTP) — all processes relevant to both cortical and retinal signaling. Furthermore, recent structural studies using cryo-EM and mutagenesis have emphasized the importance of the C-terminal PDZ-binding motif in PSD95 anchoring, and of the extracellular β1–β2 loop in mediating allosteric modulation of AMPAR gating [69], [70].

Our results also contribute to the broader understanding of how accessory subunits like TARPs, previously underappreciated in clinical genomics, can act as disease modulators rather than primary culprits — shaping the penetrance and expressivity of known pathogenic alleles. This concept is especially relevant in the context of oligogenic inheritance, where the total genetic load determines disease onset and severity, as seen in other IRDs such as Bardet-Biedl syndrome or digenic retinitis pigmentosa [71], [72].

The convergence of data from *in silico*, structural modeling, and experimental morpholino-based validation in zebrafish strengthens the notion that *CACNG8* loss or malfunction — particularly through variants affecting the PDZ domain and TM helices — impairs AMPAR tethering to postsynaptic densities and alters ionotropic glutamate receptor signaling in the retina. This has potential implications not only for IRDs but also for broader neurodevelopmental and neurodegenerative conditions where AMPAR dysregulation is implicated, including epilepsy, schizophrenia, and Alzheimer’s disease [73], [74].

Thus, this work positions *CACNG8* as a central node at the intersection of genetic, structural, and synaptic networks — offering a new lens through which to interpret phenotypic variability in IRDs and to refine our understanding of glutamatergic transmission in visual circuits.

### 4.2 Effects of *CACNG8* variants on AMPAR–TARP complex stability

The functional integrity of AMPAR–TARP complexes is intimately linked to the spatial and biochemical stability of the interactions between the ionotropic AMPA receptor core (composed of GRIA subunits) and auxiliary proteins such as TARP γ-8. Our structural and molecular dynamics-based analyses offer critical insight into how specific mutations in *CACNG8* — notably Leu96Val, Val102Met, Val146Gly, and the Arg123Ter truncation — differentially influence the assembly and stability of these complexes.

Through homology modeling and energy-minimized docking, all 14 complexes involving wild-type and mutant forms of TARP γ-8 were evaluated for changes in binding free energy (ΔGbind), interface area, root-mean-square deviation (RMSD), and hydrogen bonding networks. Among them, the wild-type complex (Cacng8_wild_gria1_4_psd95_cnih2_3) demonstrated the lowest ΔGbind values (−11.2 kcal/mol), the most extensive interfacial surface (∼1896 Å²), and a stable interaction profile with 8–10 hydrogen bonds consistently maintained during equilibrium-phase MD simulations. These values served as the baseline for comparative interpretation.

Variants such as Leu96Val, predicted to be hypomorphic based on prior genomic annotations, preserved near-wild-type interaction profiles. Its ΔGbind remained favorable (−10.8 kcal/mol), with minimal perturbation to key β1–β2 loop positioning or PDZ domain orientation. These data support the classification of Leu96Val as a functional conservative mutation, unlikely to disrupt AMPAR gating or receptor tethering under physiological conditions.

In contrast, the Val102Met and Val146Gly variants exhibited moderate structural instability. Both showed increased interface RMSD (2.1–2.4 Å), partial loss of hydrogen bonds, and altered loop positioning, especially in the extracellular β4–TM2 segment, which is crucial for ligand-binding domain (LBD) stabilization. The Val102Met variant in particular resulted in weaker interaction with GRIA1, compromising the interface compactness and reducing the binding energy to −9.1 kcal/mol.

However, the most deleterious effect was associated with the Arg123Ter nonsense variant, which leads to truncation of the entire cytoplasmic tail of TARP γ-8, including the PDZ-binding motif essential for PSD95 recruitment. Structural modeling revealed complete loss of interaction with PSD scaffolding proteins, a drastic reduction in interface area (∼843 Å²), and a ΔGbind of only −6.4 kcal/mol. These findings reflect a functionally null interaction, in line with the hypothesis of this variant acting as a driver of AMPAR misregulation.

Further RMSF analyses confirmed increased flexibility and disorder in the truncated and double-mutant complexes, especially in the membrane-adjacent intracellular helices, likely impairing their ability to stabilize AMPAR at postsynaptic densities. The simulation-derived conformers also revealed an increased tendency toward open-channel desensitization states in GRIA1-containing complexes paired with Arg123Ter and Val146Gly variants.

Collectively, our data underline that AMPAR–TARP complex stability is finely modulated by the structural integrity of specific extracellular and intracellular motifs in TARP γ-8. Variants that impair loop folding (Val102Met), disrupt PDZ-domain anchoring (Arg123Ter), or compromise helix–helix interactions (Val146Gly) severely alter the dynamic equilibrium of the receptor–auxiliary complex. This mechanistic understanding reinforces the role of *CACNG8* as a modifier gene and clarifies how different allelic configurations may yield a gradient of functional disruption at the molecular level.

### 4.3 Implications for synaptic plasticity and modifier gene action

The present findings provide strong evidence that *CACNG8*, encoding the Transmembrane AMPAR Regulatory Protein γ-8 (TARP γ-8), exerts a significant influence on synaptic plasticity and neurotransmission, positioning it as a potent modifier gene in the genetic architecture of inherited retinal dystrophies (IRDs). Beyond its canonical expression in the hippocampus, where it regulates AMPA receptor gating and long-term potentiation (LTP), TARP γ-8 is also enriched in retinal ganglion cells and interneurons, suggesting a broader neurophysiological role that encompasses the visual system.

The observed phenotypic manifestations in evaluated patients—particularly those carrying truncating or structurally destabilizing *CACNG8* variants—reveal a synaptopathic profile in which photoreceptor cells are preserved to varying degrees, yet the transmission of visual signals to higher-order neurons is compromised. This aligns with TARP γ-8’s known function in stabilizing AMPARs at the synaptic membrane and facilitating efficient glutamatergic transmission via its interactions with PSD-95 and other scaffolding proteins.

From a mechanistic perspective, our molecular docking and dynamics simulations delineate how specific mutations impair TARP γ-8’s interaction with key AMPA receptor subunits (notably GRIA1–4) and disrupt essential conformational motifs (e.g., the β1–β2 loop, transmembrane helices TM3 and TM4, and the PDZ-binding domain at the C-terminus). The truncating Arg123Ter variant, in particular, results in a premature stop codon that eliminates domains required for both membrane integration and postsynaptic anchoring, thereby abolishing TARP’s auxiliary function. Functionally, this likely prevents synaptic retention of AMPARs and impairs receptor trafficking, desensitization recovery, and channel conductance properties—core processes underlying LTP and synaptic adaptability.

The impact of hypomorphic alleles such as Leu96Val, which preserves most structural and interaction properties, further illustrates the concept of *modifier gene action*. In individuals carrying pathogenic variants in canonical IRD genes (e.g., *GRIA1*, *CNGB3*, *RHO*), the presence of specific *CACNG8* alleles may modulate disease severity, age of onset, or even phenotypic expression. This is evident in our familial data, where segregating combinations of *CACNG8* and IRD gene variants correlated with distinct clinical outcomes, despite similar backgrounds.

Importantly, these findings echo broader trends in neurogenetics and complex disorders, where the penetrance and expressivity of primary mutations are increasingly recognized as being shaped by a polygenic landscape involving both direct interactors and pathway-level modifiers. In the case of TARP γ-8, its role at the synaptic interface—modulating receptor conformation, interaction networks, and glutamate availability—provides a biologically plausible mechanism through which it can potentiate or mitigate disease phenotypes.

This concept is further supported by prior studies in epilepsy, schizophrenia, and autism spectrum disorders, where auxiliary subunits of ionotropic receptors (including TARPs) have been implicated in synaptic noise filtering, gain modulation, and circuit-level stability. Our data extend this paradigm into the retina, suggesting that synaptic plasticity disruptions may underlie atypical visual phenotypes and modify disease trajectories in retinal degeneration.

Thus, *CACNG8* emerges not merely as a passive modifier, but as a dynamic orchestrator of synaptic integrity, whose genetic disruption can act as a switch influencing the functional outcome of inherited mutations in IRD genes.

### 4.4 Limitations and future perspectives

Despite the integrative and multilayered design of this study—spanning human genetics, *in vivo* functional models, and state-of-the-art computational approaches—some methodological limitations must be acknowledged to properly contextualize the findings and inform future research directions.

Firstly, WES enabled the identification of potentially causative or modifier variants across multiple families, its inherent inability to capture deep intronic, promoter, enhancer, and non-coding regulatory elements may result in the underestimation of *CACNG8*-associated allelic diversity and regulatory mutations. Whole-genome sequencing (WGS), combined with transcriptomic and epigenomic profiling, would be necessary to comprehensively define the regulatory architecture influencing *CACNG8* expression and function, especially in retinal and hippocampal tissues.

Secondly, *in vivo* experiment on animal models, e. g. using CRISPR/Cas9 knock-in mouse or zebrafish models carrying the human *CACNG8* mutations (e.g., Arg123Ter or Val102Met) will be critical to dissect context-specific neuronal phenotypes in retinal and hippocampal circuits.

Thirdly, the molecular docking and molecular dynamics simulations were based on homology-modeled complexes, given the absence of high-resolution structures for full-length *CACNG8* in complex with AMPAR subunits in a mammalian synaptic context. Although structural templates from cryo-EM studies (e.g., [7], [70]) were rigorously employed, the flexibility of unstructured terminal regions (notably the C-terminal PDZ-binding domain) and the dynamic nature of the lipid environment limit structural resolution. As a result, the precise positioning of key loops (e.g., β1–β2, TM3–TM4 linkers) during synaptic docking events remains hypothetical.

Another important consideration pertains to MM/PBSA and MM/GBSA binding free energy calculations, which rely on implicit solvation models and do not account for conformational entropy unless supplemented with additional calculations, which were not performed in this study. Although radial distribution functions (RDFs) and GIST maps partially addressed this issue, future refinement with enhanced sampling methods such as metadynamics or free energy perturbation (FEP) simulations may yield more quantitative insights.

Furthermore, a notable limitation of this study is the reliance on a single MD replica per protein–ligand system. Although simulations were extended to 200–500 ns and convergence metrics were assessed, the lack of replicates may limit conformational sampling, particularly for dynamic or flexible complexes. This limitation also affects kinetic projections such as TICA, which are sensitive to trajectory diversity.

Lastly, while evidence of *CACNG8* modifier role was supported by co-segregation patterns and molecular interaction analyses, the precise thresholds by which its variants influence IRD phenotypes remain to be systematically defined. Longitudinal clinical studies across larger cohorts—paired with electrophysiological, imaging, and psychophysical readouts—will be crucial to define genotype–phenotype correlations and validate *CACNG8* predictive potential as a modifier.

Finally, while segregation and structural data are consistent with a modifier gene effect, the absence of statistical validation across larger independent cohorts limits definitive classification of *CACNG8* as a genetic modifier. Therefore, our findings should be considered hypothesis-generating and require replication in broader datasets.

Despite the consistency of our computational findings, we acknowledge that the absence of experimental validation currently limits the translational interpretation of variant effects. Future work will be required to assess these predictions via electrophysiological assays, AMPAR trafficking studies, or co-immunoprecipitation of wild-type versus mutant TARP–receptor complexes.

Thus, to expand upon the foundations laid by this work, future studies should aim to:

• Employ genome editing in human induced pluripotent stem cells (iPSCs) to create *CACNG8*-mutant retinal organoids, offering an *ex vivo* system to track synaptic maturation and degeneration.
• Integrate single-cell RNA-seq data from human retina to map the cell-type-specific expression dynamics of *CACNG8* and its interaction partners under physiological and pathological conditions.
• Extend the computational framework to simulate ternary or quaternary complexes involving *CACNG8*, AMPAR subunits, and postsynaptic scaffold proteins (e.g., PSD-95, SHANK3), incorporating lipid membranes and native ionic gradients.
• Explore the pharmacological modulation of TARP-AMPAR interactions as a therapeutic avenue, particularly for patients harboring partial loss-of-function variants amenable to functional rescue.

These directions, taken together, will enhance the resolution at which *CACNG8* synaptic role is understood and lay the groundwork for its inclusion in precision medicine workflows for IRDs and potentially broader synaptopathies.

## 5 Conclusion

This study presents a comprehensive, multi-scale investigation into the role of *CACNG8*, encoding the Transmembrane AMPA Receptor Regulatory Protein γ-8 (TARP γ-8), as a potential modifier gene in inherited retinal dystrophies (IRDs). By integrating human genetic data and computational structural analyses—including docking, molecular dynamics, MM-PBSA, GIST, and advanced residue-level profiling—we provide strong evidence that *CACNG8* contributes to synaptic dysfunction in the retina and beyond.

Across multiple affected families, we identified *CACNG8* variants—including Arg123Ter, Leu96Val, Val102Met, and Val146Gly—in compound or modifying configurations with other IRD-associated mutations. These were associated with a spectrum of phenotypes ranging from cone-rod dystrophy to optic nerve atrophy, not fully explained by known causative alleles alone.

Docking studies and Molecular Dynamics simulations revealed distinct alterations in protein–protein interaction energetics and structural geometry across 14 modeled TARP γ-8 complexes. Truncating mutations like Arg123Ter abolished key transmembrane and PDZ-binding regions, disrupting anchoring to PSD95 and AMPARs. Conversely, Leu96Val showed near-wild-type structural and energetic behavior, supporting its classification as a hypomorphic variant.

Advanced solvation and free energy analyses using GIST and MM-PBSA/MM-GBSA underscored how certain mutations alter local hydration landscapes and compromise ion–ligand coupling at synaptic interfaces. Simultaneously, IFP, pocket occupancy, and TICA/PCA analyses revealed conformational instability and reduced ligand accessibility in several mutant complexes.

Taken together, these findings support a critical regulatory role for CACNG8 in synaptic AMPAR assembly, gating, and stabilization, extending its relevance from central glutamatergic transmission to retinal excitatory synapses. The evidence presented here positions *CACNG8* not only as a plausible genetic modifier in IRDs, but also as a potential target for therapeutic modulation in diseases marked by AMPAR dysfunction.

Future investigations employing genome-edited iPSC-derived retinal organoids, multi-omic integration, and pharmacological modulation of TARP–AMPAR interactions may further delineate the translational relevance of our findings and pave the way for personalized therapeutic strategies.

## CRediT authorship contribution statement

**Luigi Donato:** Writing – review & editing, Writing – original draft, Conceptualization. **Lucia Poggi:** Writing – original draft, Formal analysis, Data curation. **Concetta Scimone:** Formal analysis, Data curation. **Scalinci Segio Zaccaria:** Resources, Funding acquisition. **Domenico Mordà:** Formal analysis, Data curation. **Rosalia D’Angelo:** Writing – review & editing, Conceptualization. **Simona Alibrandi:** Writing – original draft, Formal analysis, Data curation. **Antonina Sidoti:** Writing – review & editing, Conceptualization. **Ignacio Babiloni Chust:** Formal analysis, Data curation. **Carmela Rinaldi:** Formal analysis, Data curation. **Giorgia Abate:** Formal analysis, Data curation.

## Ethics statement

This study was approved by the ethics committee of the “Azienda Policlinico Universitario di Messina” (approval number: 23/17bis, prot. n. 0014661). All procedures involving human participants were performed in accordance with institutional and/or national research committee ethical standards and performed in accordance with the 1964 Declaration of Helsinki and its later amendments or comparable ethical standards. The participants gave written informed consent for the publication of any images, clinical data and other data included in the manuscript.

## Funding

This research received no external funding.

## Declaration of Competing Interest

The authors declare that they have no known competing financial interests or personal relationships that could have appeared to influence the work reported in this paper.

## Data Availability

The data are available as supplementary materials and upon request. Input files and scripts used in docking and molecular dynamics simulations will be made available upon final publication through a public repository.

## Footnotes

Appendix A Supplementary data associated with this article can be found in the online version at doi:10.1016/j.csbj.2025.09.038.
