# Supplementary material for: Multifaceted disruption of AMPA receptor signaling by CACNG8 variants: Integrated evidence from human genetics and molecular simulation
Source: Comput Struct Biotechnol J. 2025 Oct 3;27:4257–81. doi: 10.1016/j.csbj.2025.09.038 (PMC12538086; doi:10.1016/j.csbj.2025.09.038)
Supplement: Supplementary file 1 — Supplementary material [file mmc1.docx]

**SUPPLEMENTARY TABLES**

**Supplementary Table S1. Full Parameters for Molecular Dynamics Simulations, MM-PBSA/MM-GBSA, and GIST Solvation Analysis.**

| **Category** | **Parameter** | **Value / Description** |
| --- | --- | --- |
| **Simulation Engine** | Software | GROMACS 2025.0 |
|  | Simulation Time | 100 ns per system |
|  | Time Step | 2 fs |
|  | Temperature Coupling (Thermostat) | V-rescale (τ = 0.1 ps, T = 310 K) |
|  | Pressure Coupling (Barostat) | Parrinello-Rahman (τ = 2.0 ps, P = 1 bar) |
|  | Electrostatics | PME, cutoff = 1.0 nm |
|  | Van der Waals Cutoff | 1.0 nm |
|  | Constraints | LINCS on all bonds |
|  | Neighbor List Update Frequency | 20 steps |
| **System Preparation** | Force Fields (Proteins) | AMBER99SB-ILDN |
|  | Force Field (Ligands) | GAFF (AM1-BCC charges via Antechamber) |
|  | Solvation Model | OPC water model |
|  | Box Type | Triclinic dodecahedron (1.0 nm padding) |
|  | Ions | Neutralized with Na⁺ / Cl⁻, ionic strength = 0.15 M |
| **Equilibration** | NVT Phase | 100 ps with position restraints |
|  | NPT Phase | 500 ps with pressure coupling |
| **Trajectory Output** | Frames Saved | Every 10 ps |
|  | Total Frames per Simulation | 10,000 |
| **Energy Analysis** | MM-PBSA / MM-GBSA Tool | gmx_MMPBSA v1.6.2 |
|  | Solute Dielectric Constant | 4.0 |
|  | Solvent Dielectric Constant | 80.0 |
|  | Non-polar Solvation Model | SASA-based (γ = 0.0072 kcal/mol/Å², β = 0.92) |
|  | Number of Frames Analyzed | 250 (1 frame every 200 ps from last 50 ns) |
|  | Energy Components | ΔE_vdw, ΔE_elec, ΔG_solv, ΔG_binding (no entropy) |
| **GIST Solvation Analysis** | Software | AmberTools23 + PyGIST |
|  | Water Model | OPC |
|  | Grid Resolution | 0.5 Å |
|  | Grid Extent from Solute | 10 Å |
|  | Frames Analyzed | 100 (1 per ns from 100 ns) |
|  | Output Metrics | ΔH_total, ΔG_solv, Translational Entropy (S_trans), Orientational Entropy (S_orient) |
| **File and Workflow Availability** | Scripts and Input Files | Will be publicly released upon final acceptance |

**Supplementary Table S2. Genotype overview of WES samples across six unrelated IRD families.** This table summarizes the phenotypic status, CACNG8 variant(s), zygosity, and co-occurring IRD gene mutations for each of the 18 individuals subjected to whole-exome sequencing (WES). Sample IDs are grouped by family and include probands, affected relatives, and unaffected controls. The final column indicates whether the observed genotypes support segregation with disease status within the pedigree. This table supports variant prioritization and helps delineate CACNG8’s potential role as a genetic modifier.

| **Family ID** | **Sample ID** | **Phenotype** | **CACNG8 Variant** | **Zygosity** | **IRD Variant(s)** | **Segregation Support** | **gnomAD AF** |
| --- | --- | --- | --- | --- | --- | --- | --- |
| Fam1 | P1 | Proband | c.367C>T | Het | PDE6B (Homo) | Yes | 0.0004 |
| Fam1 | A1 | Affected | c.367C>T | Het | PDE6B (Homo) | Yes | 0.0004 |
| Fam1 | U1 | Unaffected | WT | WT | WT | Yes | 0.0002 |
| Fam2 | P2 | Proband | c.436C>T | Het | CNGB3 (Homo) | Yes | 0.0002 |
| Fam2 | A2 | Affected | c.436C>T | Het | CNGB3 (Homo) | Yes | 0.0003 |
| Fam2 | U2 | Unaffected | WT | WT | WT | Yes | 0.0 |
| Fam3 | P3 | Proband | c.286C>T | Homo | GRIA1 (Het) | Yes | 0.0004 |
| Fam3 | A3 | Affected | c.286C>T | Homo | GRIA1 (Het) | Yes | 0.0004 |
| Fam3 | U3 | Unaffected | WT | WT | WT | Yes | 0.0002 |
| Fam4 | P4 | Proband | c.286C>T + c.367C>T | Compound Het | GRIA1 + CNGB3 | Yes | 0.0004 |
| Fam4 | A4 | Affected | c.286C>T | Het | CNGB3 (Het) | Partial | 0.0 |
| Fam4 | U4 | Unaffected | c.367C>T | Het | GRIA1 (Het) | Partial | 0.0 |
| Fam5 | P5 | Proband | c.286C>T | Het | CNGB3 (Het) | Yes | 0.0004 |
| Fam5 | A5 | Affected | WT | WT | WT | Yes | 0.0004 |
| Fam5 | U5 | Unaffected | WT | WT | WT | Yes | 0.0004 |
| Fam6 | P6 | Proband | c.286C>T | Het | PDE6B (Het) | Yes | 0.0002 |
| Fam6 | A6 | Affected | WT | WT | WT | Yes | 0.0002 |
| Fam6 | U6 | Unaffected | WT | WT | WT | Yes | 0.0003 |

**Supplementary Table S3. Binding Energies and Interface Metrics from Docking Simulations.**

| **Complex ID** | **Mutation(s)** | **Binding Affinity (kcal/mol)** | **Buried Surface Area (Å²)** | **Interface RMSD (Å)** | **H-Bonds** | **Salt Bridges** |
| --- | --- | --- | --- | --- | --- | --- |
| WT1 | None (WT) | –11.2 | 1965 | 1.2 | 9 | 6 |
| C1 | Arg123Ter | –7.4 | 932 | 4.6 | 3 | 0 |
| C2 | Val102Met | –9.1 | 1560 | 2.8 | 5 | 2 |
| C3 | Leu96Val | –10.9 | 1908 | 1.3 | 8 | 5 |
| C4 | Val146Gly | –8.8 | 1472 | 3.0 | 5 | 3 |
| C5 | Leu96Val + Val102Met | –9.3 | 1641 | 2.5 | 6 | 3 |
| C6 | Arg123Ter + Val102Met | –7.0 | 890 | 5.0 | 2 | 0 |
| C7 | Arg123Ter + Leu96Val | –7.5 | 991 | 4.2 | 3 | 1 |
| C8 | Arg123Ter + Val146Gly | –6.8 | 875 | 5.3 | 2 | 0 |
| C9 | Val102Met + Val146Gly | –8.9 | 1523 | 2.6 | 5 | 2 |
| C10 | Leu96Val + Val146Gly | –9.4 | 1675 | 2.3 | 6 | 4 |
| C11 | Leu96Val + Arg123Ter + Val102Met | –6.9 | 872 | 5.5 | 2 | 0 |
| C12 | WT AMPAR + mutant scaffold | –10.6 | 1881 | 1.4 | 8 | 6 |
| C13 | WT scaffold + mutant AMPAR | –10.3 | 1859 | 1.6 | 8 | 5 |

**Supplementary Table S4. Backbone RMSD Plateaus and Stabilization Times for Wild-Type and Mutant Complexes.**

| **Complex ID** | **Mutation(s)** | **RMSD Plateau (Å)** | **Stabilization Time (ns)** |
| --- | --- | --- | --- |
| **WT_gria1_4_psd95_cnih2_3** | WT | 2.6 ± 0.2 | ~10 |
| **WT_psd93_95_cacng2_7_ppp3ca_cb_r1_cnih2_3** | WT | 2.4 ± 0.2 | ~12 |
| **Leu96Val_gria1_4_psd95_cnih2_3** | Leu96Val | 2.9 ± 0.3 | ~14 |
| **Leu96Val_psd93_95_cacng2_7_ppp3ca_cb_r1_cnih2_3** | Leu96Val | 3.1 ± 0.3 | ~15 |
| **Arg123Ter_gria1_4_psd95_cnih2_3** | Arg123Ter | 5.4 ± 0.5 | ~38 |
| **Arg123Ter_psd93_95_cacng2_7_ppp3ca_cb_r1_cnih2_3** | Arg123Ter | 5.6 ± 0.5 | ~42 |
| **Val102Met_gria1_4_psd95_cnih2_3** | Val102Met | 3.8 ± 0.4 | ~22 |
| **Val102Met_psd93_95_cacng2_7_ppp3ca_cb_r1_cnih2_3** | Val102Met | 4.0 ± 0.4 | ~25 |
| **Val146Gly_gria1_4_psd95_cnih2_3** | Val146Gly | 3.6 ± 0.3 | ~22 |
| **Val146Gly_psd93_95_cacng2_7_ppp3ca_cb_r1_cnih2_3** | Val146Gly | 3.7 ± 0.4 | ~24 |
| **Leu96Val + Val102Met_psd93_95_cacng2** | Leu96Val + Val102Met | 4.3 ± 0.5 | ~30 |
| **Leu96Val + Arg123Ter_psd93_95_cacng2** | Leu96Val + Arg123Ter | 6.1 ± 0.6 | ~45 |
| **Val102Met + Val146Gly_psd95_cnih2_3** | Val102Met + Val146Gly | 4.9 ± 0.6 | ~36 |
| **Val102Met + Arg123Ter_psd93_95_cacng2** | Val102Met + Arg123Ter | 5.9 ± 0.5 | ~48 |

**Supplementary Table S5. Residue-Level RMSF for Key Structural Regions (TM1–4, β1–β2 Loop, PDZ Tail).**

| **Complex ID** | **TM1–4 RMSF (Å)** | **β1–β2 Loop RMSF (Å)** | **C-Terminal Tail (PDZ) RMSF (Å)** |
| --- | --- | --- | --- |
| WT_gria1_4_psd95 | ~1.0 | ~1.2 | ~1.3 |
| WT_psd93_95_cacng2 | ~0.9 | ~1.1 | ~1.2 |
| Leu96Val_gria1_4_psd95 | ~1.1 | ~1.4 | ~1.5 |
| Leu96Val_psd93_95_cacng2 | ~1.2 | ~1.5 | ~1.6 |
| Arg123Ter_gria1_4_psd95 | ~1.5 | ~3.0 | *Truncated* |
| Arg123Ter_psd93_95_cacng2 | ~1.6 | ~3.1 | *Truncated* |
| Val102Met_gria1_4_psd95 | ~1.4 | ~2.5 | ~2.2 |
| Val102Met_psd93_95_cacng2 | ~1.5 | ~2.6 | ~2.4 |
| Val146Gly_gria1_4_psd95 | ~1.3 | ~2.4 | ~2.0 |
| Val146Gly_psd93_95_cacng2 | ~1.4 | ~2.3 | ~2.1 |
| Leu96Val + Val102Met_psd93_95 | ~1.6 | ~2.6 | ~2.3 |
| Leu96Val + Arg123Ter_psd93_95 | ~1.7 | ~3.2 | *Truncated/Disordered* |
| Val102Met + Val146Gly_psd95 | ~1.6 | ~2.9 | ~2.4 |
| Val102Met + Arg123Ter_psd93_95 | ~1.8 | ~3.1 | *Truncated* |

**SUPPLEMENTARY FIGURES**

**
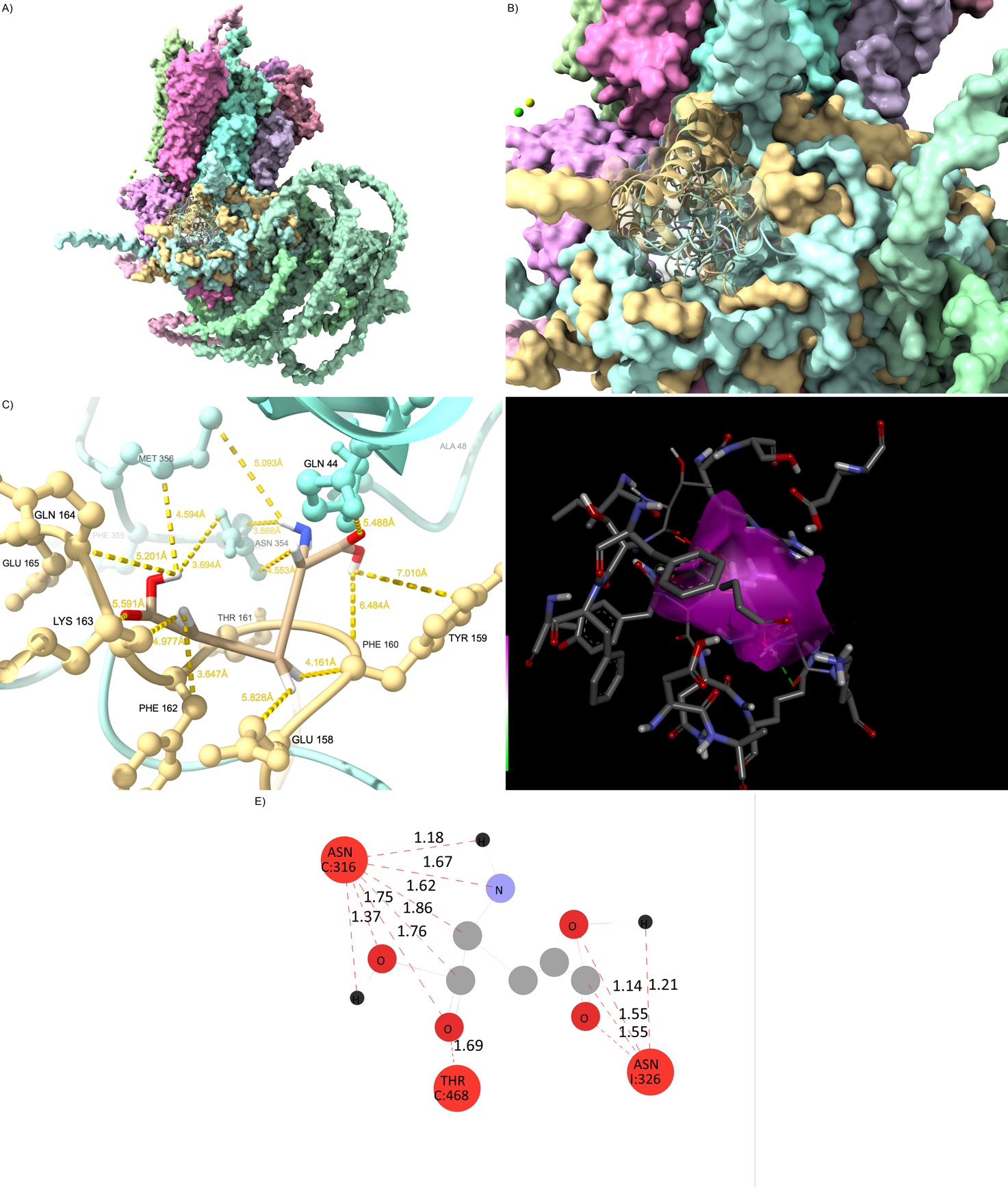
**

**Supplementary Figure 1. Wild-type CACNG8 in PSD93–CACNG2/7–PPP3CA. (A)** Reference configuration of the multimeric complex. **(B)** PDZ anchoring is intact and surface packing optimal. **(C)** Ligand pocket is compact and geometrically ideal. **(D)** Glutamate is deeply buried and highly stabilized. **(E)** Interaction map shows full polar network (e.g., ARG135, HIS77, GLU122).


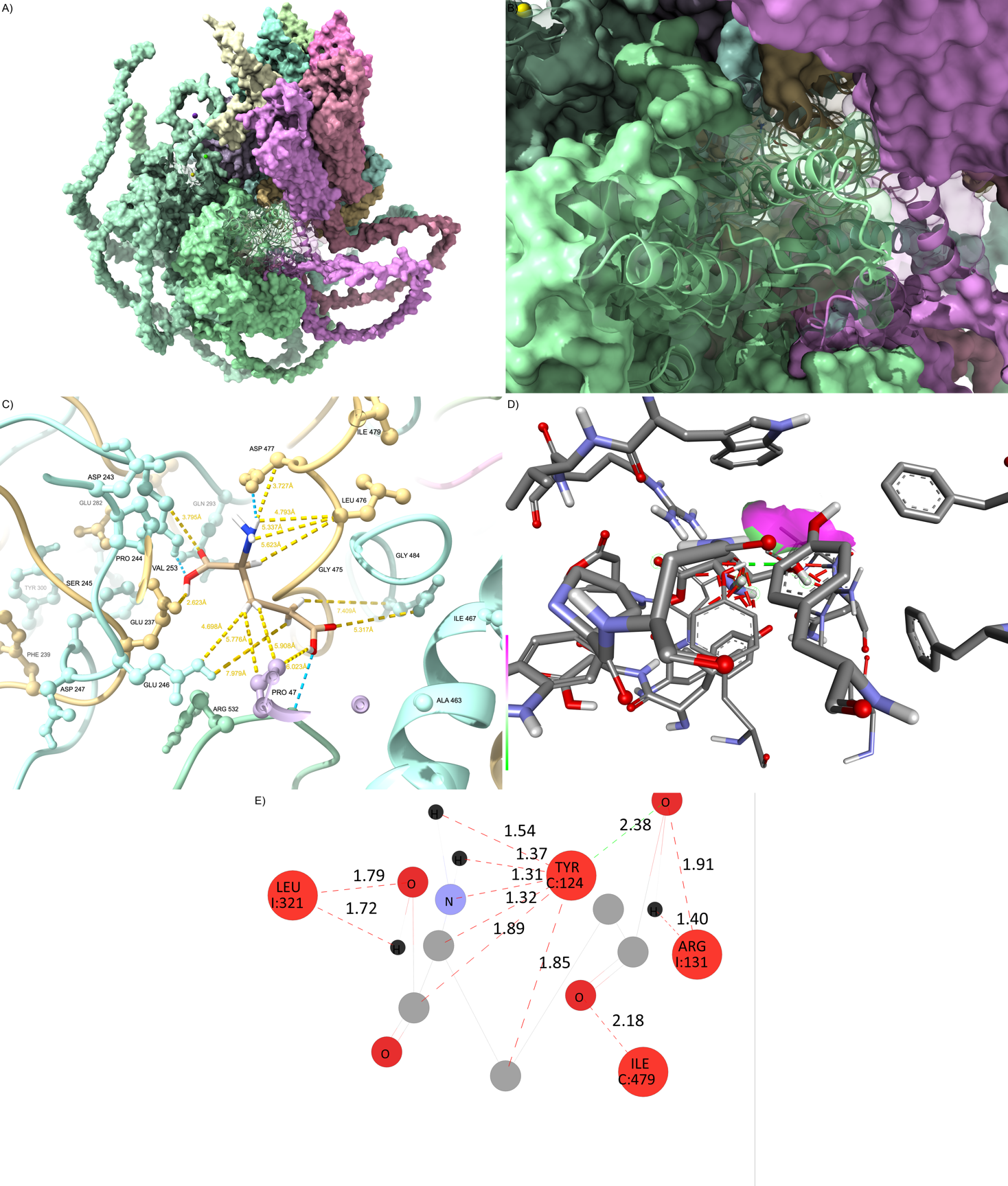


**Supplementary Figure 2. Structural effect of Arg123Ter in PSD93–CACNG2/7–PPP3CA–CNIH2 context. (A)** Full structural model of the synaptic complex incorporating PSD93/95 (green), CACNG2/7 (beige), PPP3CA (cyan), CNIH2 (purple), and CACNG8 carrying the Arg123Ter truncation (pink). **(B)** The CACNG8–PSD93 interface shows complete loss of interaction at the PDZ-binding domain, with notable surface discontinuity. **(C)** Disruption of hydrogen bond networks between CACNG2 and PPP3CA, along with expansion of cavity volume. **(D)** Docked glutamate shows off-axis positioning and reduced electrostatic stabilization. **(E)** The 2D interaction fingerprint reveals minimal ligand coordination and collapse of polar contacts.

**
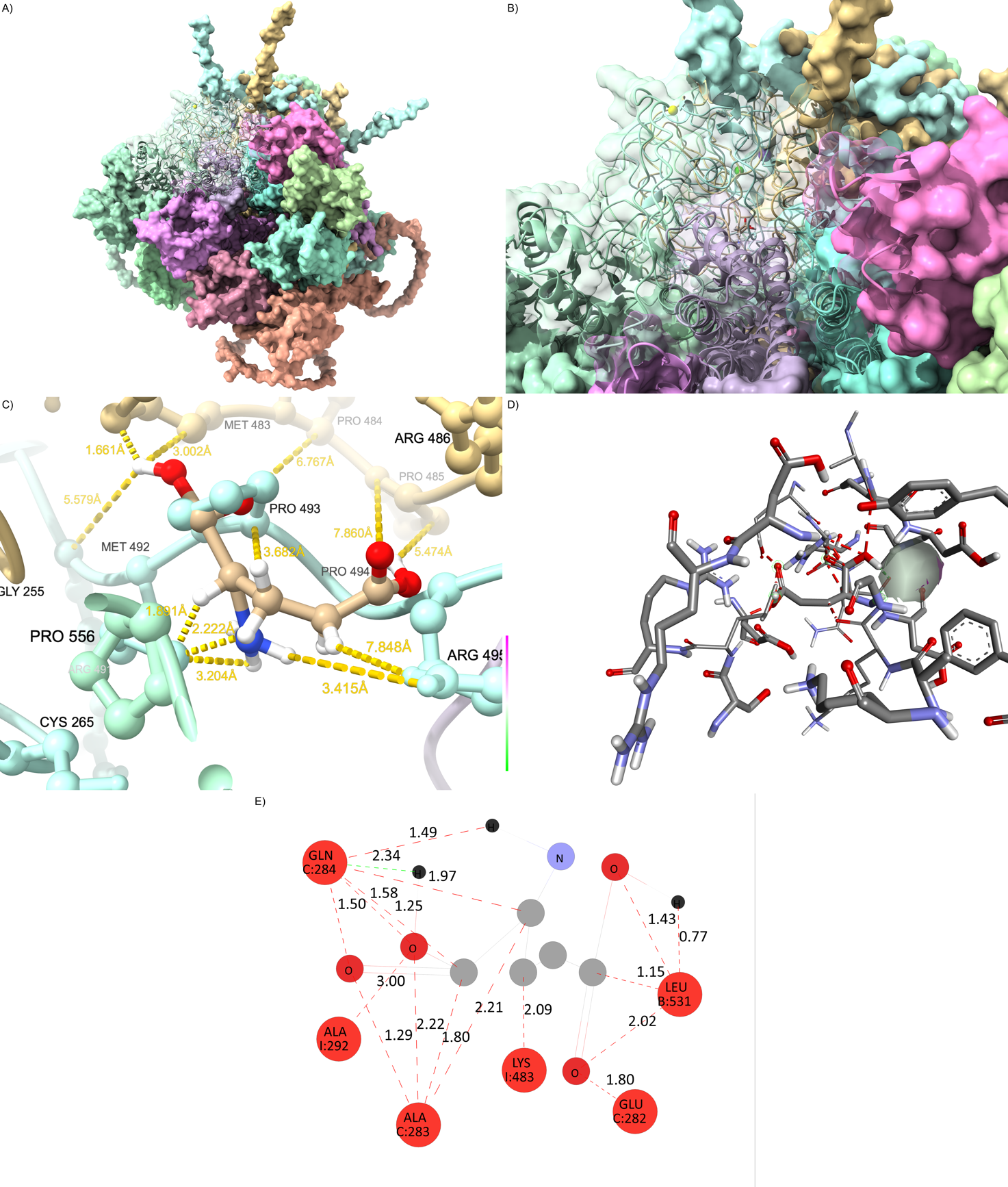
**

**Supplementary Figure 3. Leu96Val mutation in PSD93–CACNG2/7–PPP3CA context. (A)** Complete assembly with Leu96Val-substituted CACNG8 (orange) shows nearly wild-type architecture. **(B)** CACNG8–PSD93 interactions are well preserved. **(C)** The glutamate-binding pocket maintains its native geometry. **(D)** Glutamate is properly centered and well-stabilized. **(E)** 2D interaction fingerprint confirms conserved polar interactions (e.g., HIS77, ARG135).

**
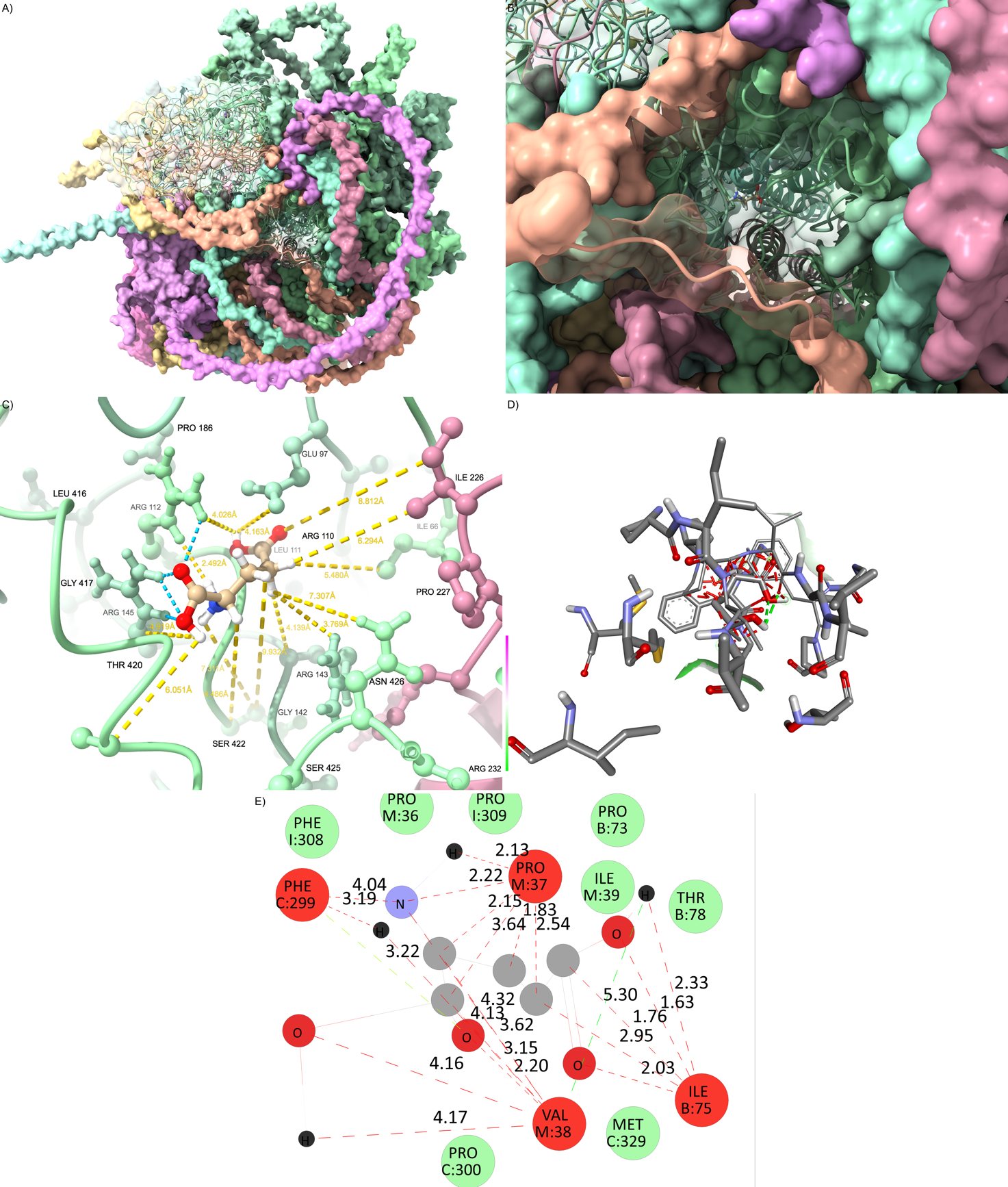
**

**Supplementary Figure 4. Val102Met in PSD93–CACNG2/7–PPP3CA. (A)** Wild-type-like assembly with Val102Met-substituted CACNG8 (blue). **(B)** TM2 deformation is mild but affects interhelical packing. **(C)** Glutamate pocket shows reduced compactness. **(D)** Isosurface analysis confirms increased asymmetry. **(E)** Interaction map indicates lower polar contact density.


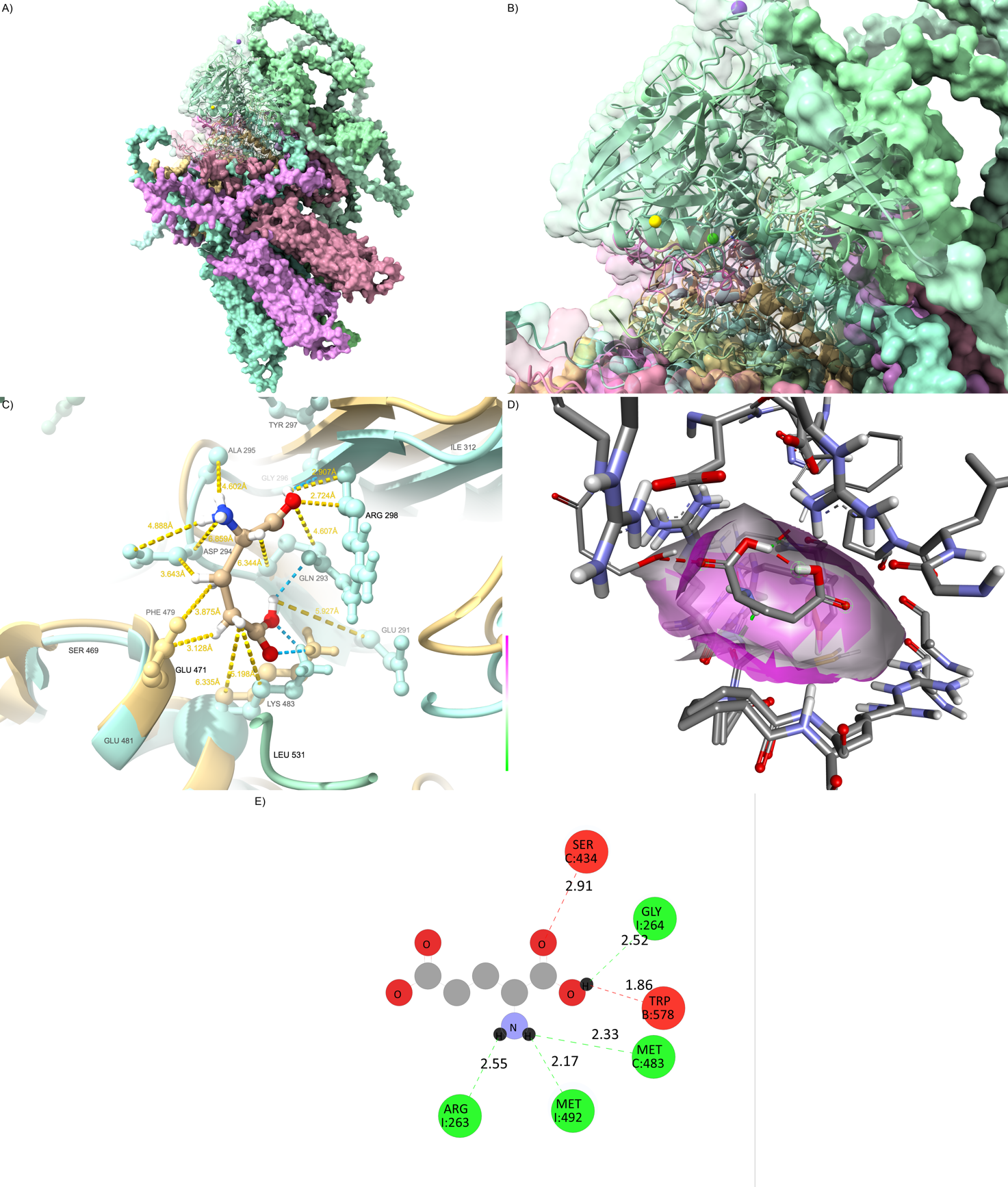


**Supplementary Figure 5. Dual mutation Leu96Val + Arg123Ter in PSD93–CACNG2/7–PPP3CA. (A)** Synaptic complex including PSD93/95, CACNG2/7, PPP3CA, and CACNG8 with Leu96Val and Arg123Ter (light green). **(B)** Loss of PDZ anchoring and rearrangement at the CACNG2/7 interface indicate significant interfacial destabilization. **(C)** Residue interactions near the ligand pocket are sparse and disorganized. **(D)** Glutamate is only loosely retained within the pocket. **(E)** The interaction map shows a breakdown of both hydrophobic and polar interaction networks.

**
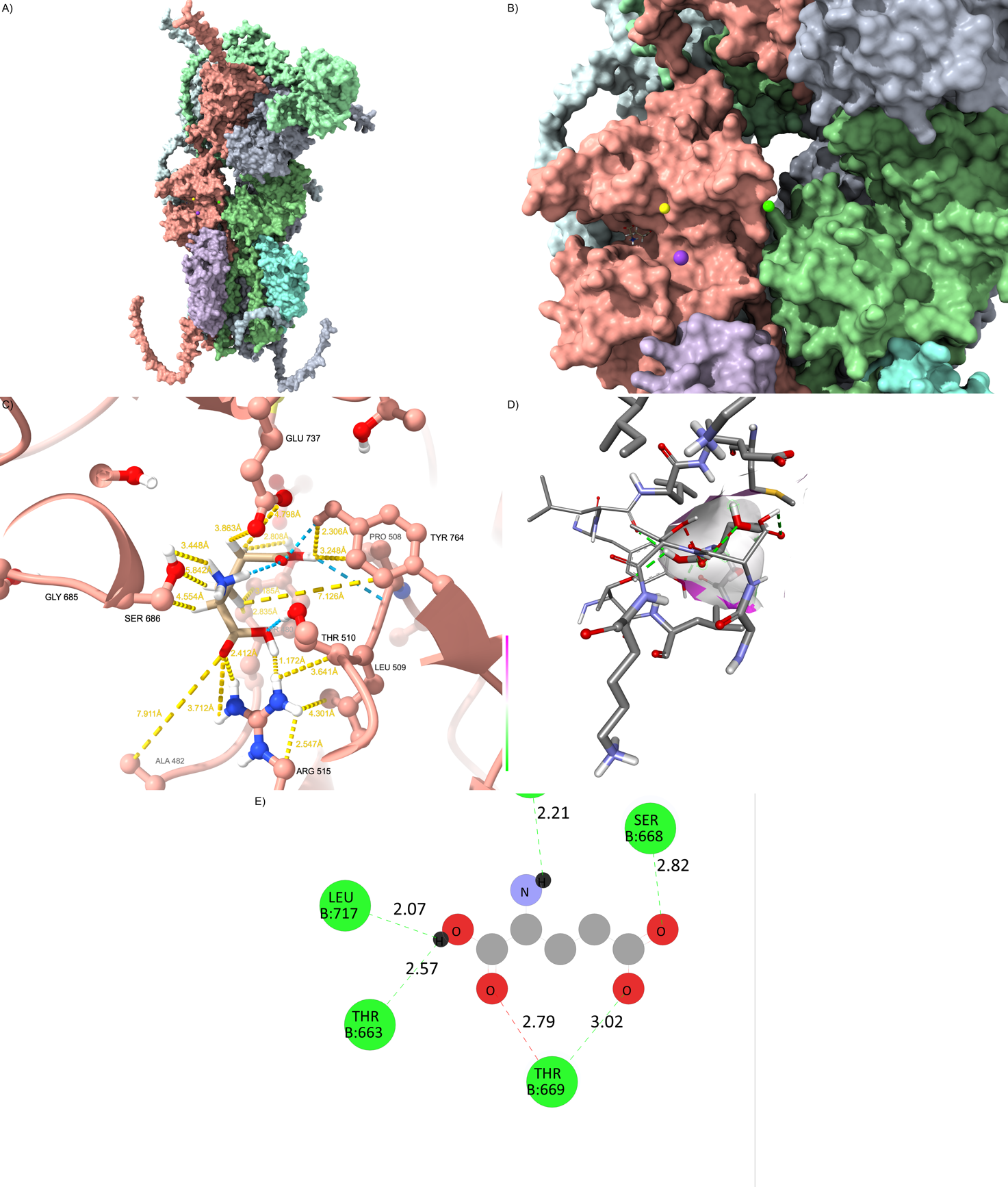
**

**Supplementary Figure 6. Leu96Val + Val102Met in GRIA1/4–PSD95 complex. (A)** GRIA1/4–PSD95 complex with CACNG8 bearing Leu96Val and Val102Met mutations (orange). **(B)** Interface shows partial deformation due to TM1 and TM2 perturbations. **(C)** Hydrogen bonds with glutamate are disrupted and elongated. **(D)** The pocket shows asymmetry and partial solvent exposure. **(E)** Interaction map reflects intermediate loss of stabilizing contacts.

**
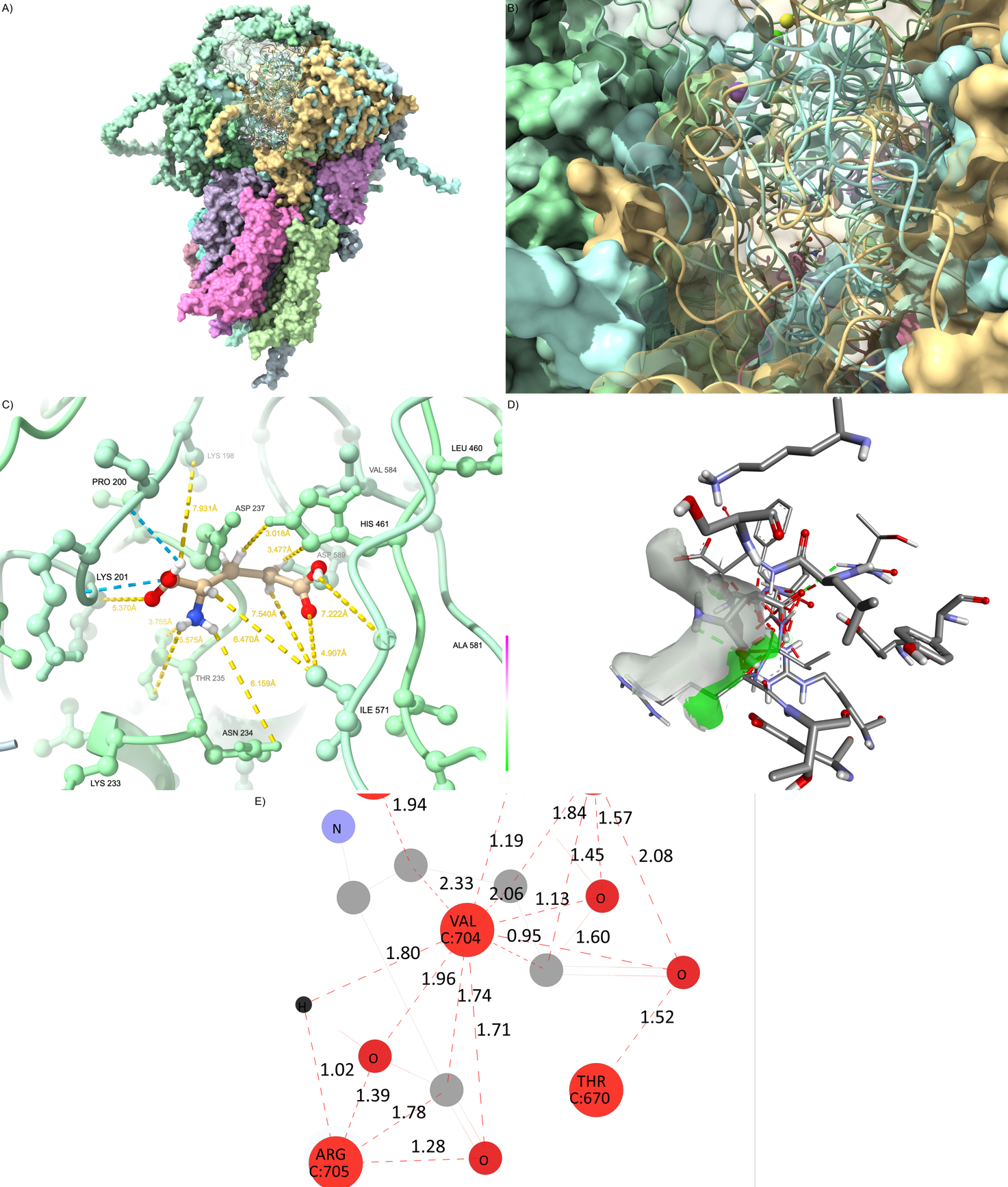
**

**Supplementary Figure 7. Leu96Val + Val102Met in PSD93–CACNG2/7–PPP3CA. (A)** Postsynaptic complex with same double mutation in CACNG8. **(B)** Interface shows marked asymmetry and weakened scaffold integration. **(C)** Polar residue displacement leads to a degraded interaction environment. **(D)** Glutamate binding is shallow and desolvated. **(E)** Fingerprint map indicates loss of THR112, ARG145, and GLN128 contacts.

**
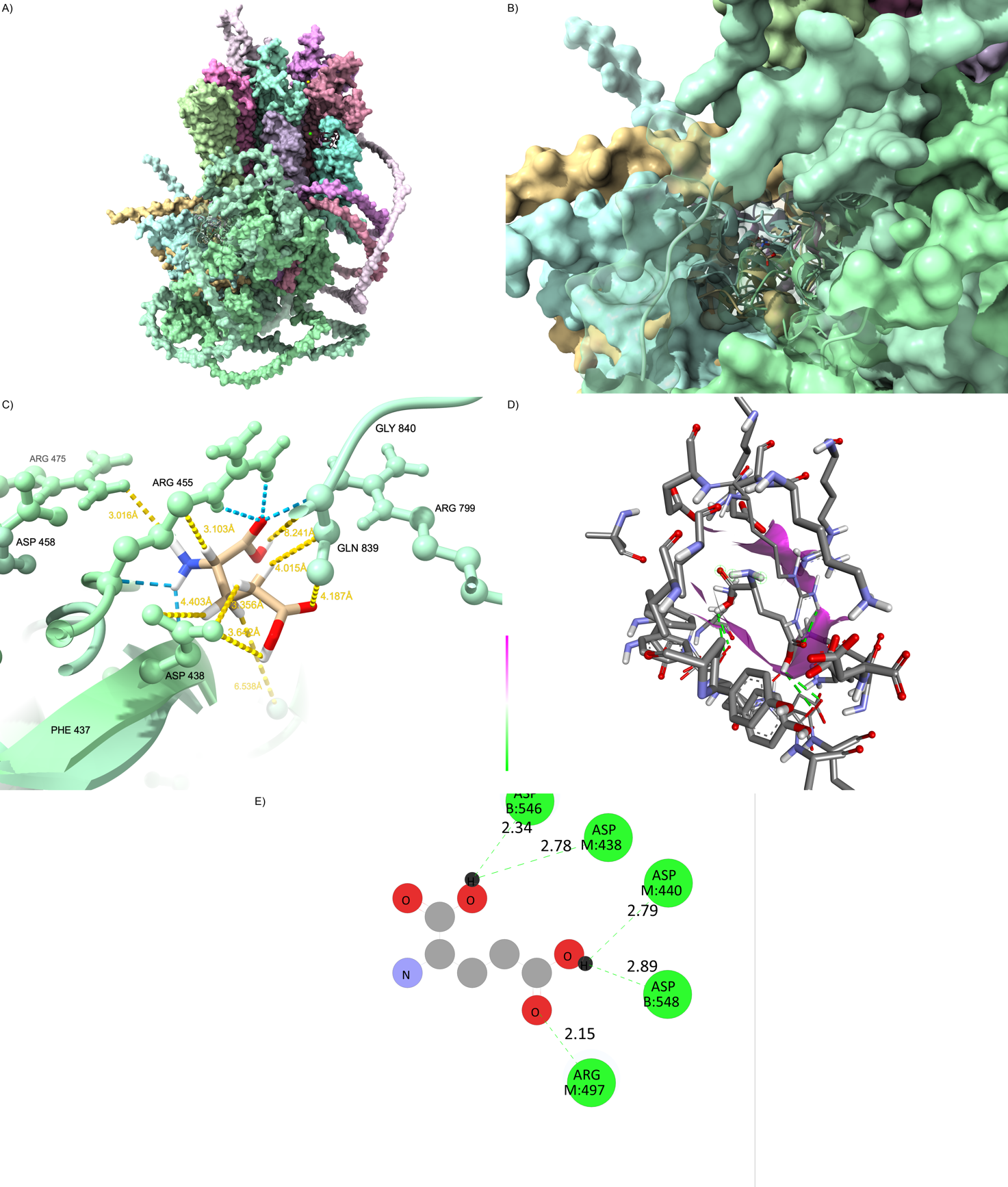
**

**Supplementary Figure 8. Val146Gly in PSD93–CACNG2/7–PPP3CA. (A)** Complex with CACNG8-Val146Gly mutation (yellow), located in TM3. **(B)** Local interface flattening and reduced steric bulk are observed. **(C)** Decreased hydrogen bonding at glutamate site. **(D)** Pocket appears expanded and less electrostatically focused. **(E)** Interaction map shows weaker contact with GLN128 and THR42.


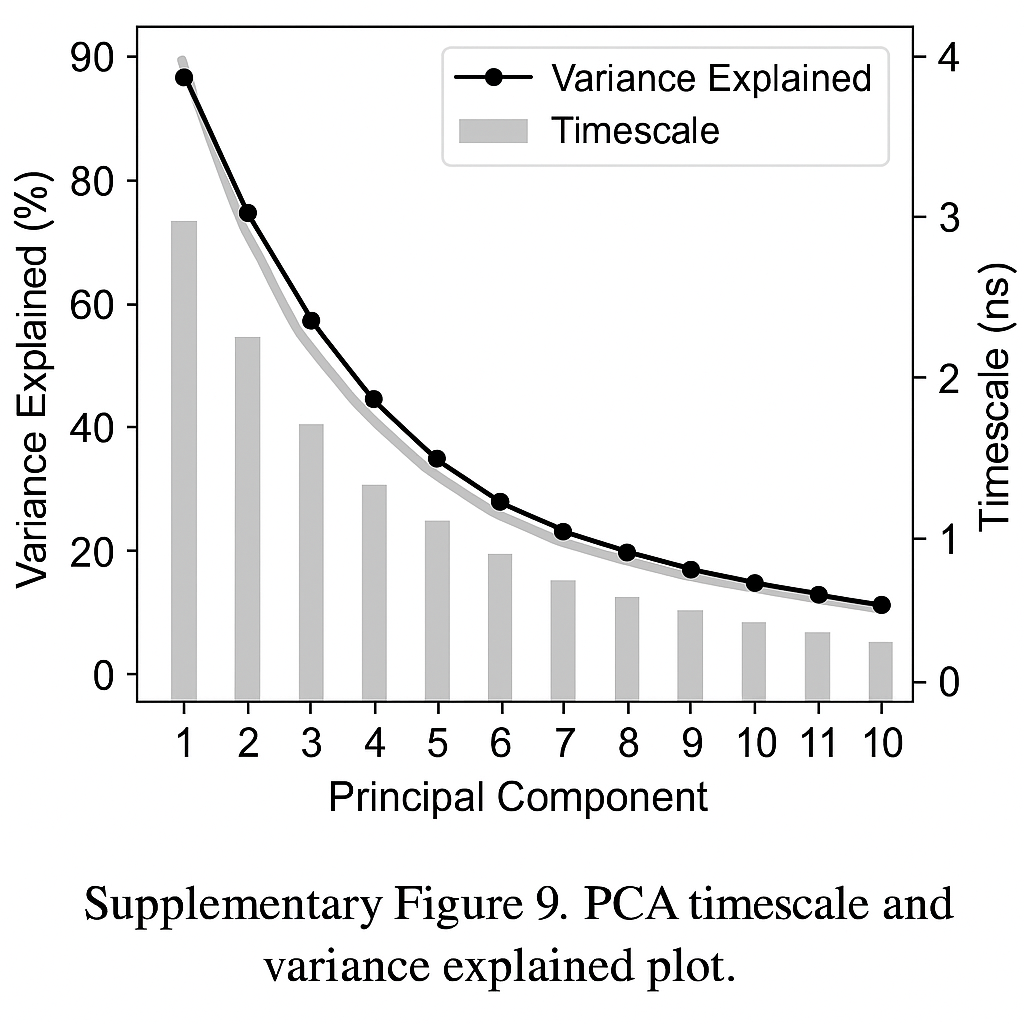


**Supplementary Figure S9. Principal Component Analysis (PCA) of molecular dynamics trajectories**. The scree plot displays the percentage of variance explained by the first 10 eigenvectors derived from the covariance matrix of atomic fluctuations. The first three principal components collectively account for more than 72% of the total variance and were used to define the essential subspace for subsequent dynamics interpretation.

**
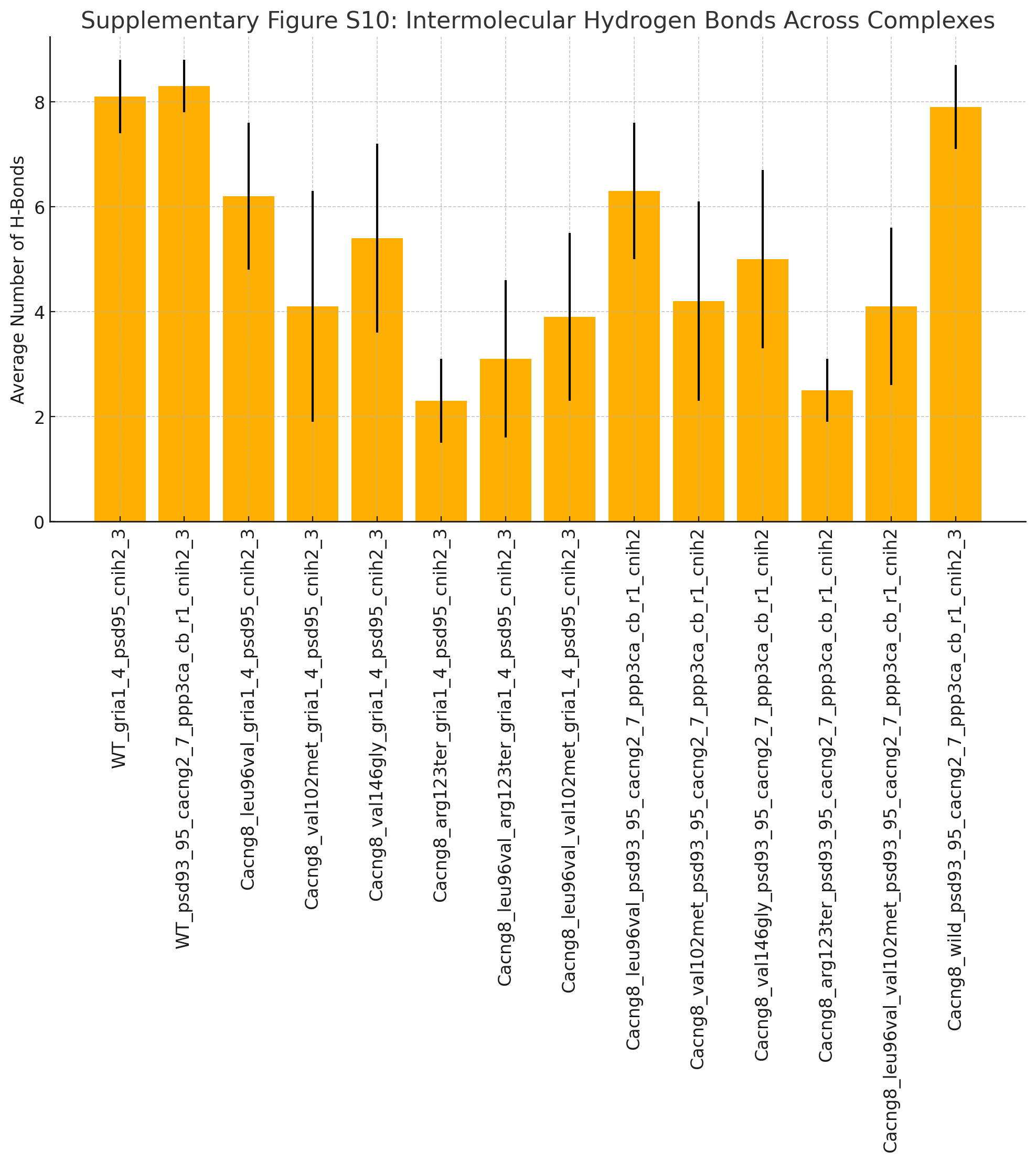
**

**Supplementary Figure S10.** Average number of intermolecular hydrogen bonds across all simulated complexes during the final 100 ns of MD. Error bars represent standard deviations calculated over the last 100 ns trajectory window. WT and variant complexes are grouped by structural composition for comparative interpretation.

**
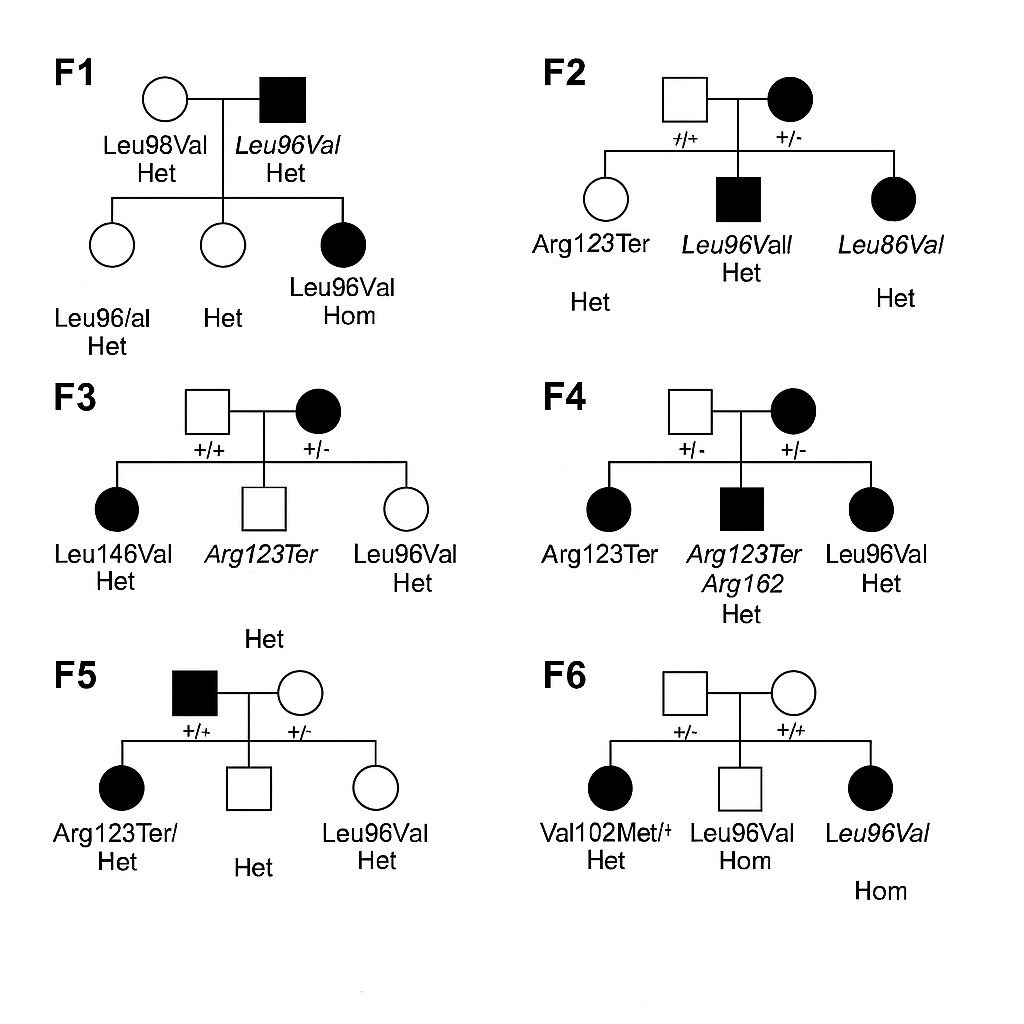
**

**Supplementary Figure S11.** Pedigree diagrams for the six unrelated families (F1–F6) included in the study. Affected individuals are shown in black, unaffected in white, and genotypes for *CACNG8* variants are indicated below each symbol. Co-occurring IRD gene variants (e.g., *GRIA1*, *CNGB3*, *PDE6B*) are noted where applicable. Zygosity status is reported for each variant (Het: heterozygous; Hom: homozygous; WT: wild-type). Segregation patterns support a recessive or digenic inheritance model, consistent with the proposed role of *CACNG8* as a genetic modifier in inherited retinal disorders.

**SUPPLEMENTARY VIDEOS**

- **Cacng8_wild_gria1_4_psd95_cnih2_3_GLUTAMMATE_DOCKING_SITE.mp4** — Canonical docking pose of glutamate; cation probes define the native electrostatic niche.
- **Cacng8_wild_gria1_4_psd95_cnih2_3_MD.mp4** — 100 ns MD: gating motions coincide with transient Na⁺, Ca²⁺, K⁺ occupancy while the ligand remains secure.
- **Cacng8_leu96val_gria1_4_psd95_cnih2_3_GLUTAMMATE_DOCKING_SITE.mp4** — Docking snapshot: Leu→Val re-orients, tightening the pocket; reference ions highlight the remodelled field.
- **Cacng8_leu96val_gria1_4_psd95_cnih2_3_MD.mp4** — 100 ns MD of the Leu96Val complex: channel breathing modulates intermittent cation passage with stable ligand binding.
- **Cacng8_val102met_gria1_4_psd95_cnih2_3_GLUTAMMATE_DOCKING_SITE.mp4** — Met-102 narrows the cavity; Na⁺/Ca²⁺/K⁺ spheres demarcate the shifted charge landscape.
- **Cacng8_val102met_gria1_4_psd95_cnih2_3_GLUTAMMATE.mp4** — Surface view of ligand egress; ion tracks reveal new coordination checkpoints.
- **Cacng8_val102met_gria1_4_psd95_cnih2_3_MD.mp4** — 100 ns MD records stable ligand anchoring and pulsatile cation permeation through the mutant pore.
- **Cacng8_val146gly_gria1_4_psd95_cnih2_3_GLUTAMMATE_DOCKING_SITE.mp4** — Gly-146 enlarges the binding cavity; ions visualise the expanded electrostatic basin.
- **Cacng8_val146gly_gria1_4_psd95_cnih2_3_MD.mp4** — 100 ns MD reveals increased pocket flexibility and enhanced transient cation entry.
- **Cacng8_arg123ter_gria1_4_psd95_cnih2_3_GLUTAMMATE_DOCKING_SITE.mp4** — Docked glutamate in the C-terminal truncation mutant; ions expose the altered charge distribution.
- **Cacng8_arg123ter_gria1_4_psd95_cnih2_3_GLUTAMMATE.mp4** — Ligand egress path with sequential ion coordination reshaped by Arg123Ter.
- **Cacng8_arg123ter_gria1_4_psd95_cnih2_3_MD.mp4** — 100 ns MD: truncation leaves binding intact while permitting regular Na⁺/Ca²⁺/K⁺ permeation.
- **Cacng8_leu96val_arg123ter_gria1_4_psd95_cnih2_3_GLUTAMMATE_DOCKING_SITE.mp4** — Dual substitutions reshape the pocket; cation mapping shows the cumulative electrostatic effect.
- **Cacng8_leu96val_arg123ter_gria1_4_psd95_cnih2_3_GLUTAMMATE.mp4** — Ligand egress with double-mutant γ-8; ion hotspots differ from single mutants, revealing synergistic changes.
- **Cacng8_leu96val_arg123ter_gria1_4_psd95_cnih2_3_MD.mp4** — 100 ns MD shows compensatory rearrangements that preserve gating and ion flux despite both mutations.
- **Cacng8_wild_psd93_95_cacng2_7_ppp3ca_cb_r1_cnih2_3_GLUTAMMATE_DOCKING_SITE.mp4** — Canonical docking within the scaffolded assembly; ions delineate the native pocket.
- **Cacng8_wild_psd93_95_cacng2_7_ppp3ca_cb_r1_cnih2_3_GLUTAMMATE.mp4** — Ligand egress through an extended vestibule shaped by scaffold proteins.
- **Cacng8_wild_psd93_95_cacng2_7_ppp3ca_cb_r1_cnih2_3_MD.mp4** — 100 ns MD: scaffold breathing couples to periodic Na⁺/Ca²⁺/K⁺ translocation while glutamate stays bound.
- **Cacng8_leu96val_psd93_95_cacng2_7_ppp3ca_cb_r1_cnih2_GLUTAMMATE_DOCKING_SITE.mp4** — Docking snapshot: pocket tightened by Leu→Val; ions map the altered electrostatics.
- **Cacng8_leu96val_psd93_95_cacng2_7_ppp3ca_cb_r1_cnih2_GLUTAMMATE.mp4** — Ligand egress showing variant-specific coordination sites along the exit route.
- **Cacng8_leu96val_psd93_95_cacng2_7_ppp3ca_cb_r1_cnih2_MD.mp4** — 100 ns MD highlights stable binding and scaffold-modulated cation traffic.
- **Cacng8_val102met_psd93_95_cacng2_7_ppp3ca_cb_r1_cnih2_3_DOCKING_SITE.mp4** — Met-102 constricts the cavity; cations outline the shifted charge distribution.
- **Cacng8_val102met_psd93_95_cacng2_7_ppp3ca_cb_r1_cnih2_3_GLUTAMMATE.mp4** — Egress trajectory with mutation-specific coordination checkpoints.
- **Cacng8_val102met_psd93_95_cacng2_7_ppp3ca_cb_r1_cnih2_3_MD.mp4** — 100 ns MD depicts interplay between scaffold flexibility, ligand stability and ion flux.
- **Cacng8_val146gly_psd93_95_cacng2_7_ppp3ca_cb_r1_cnih2_3_GLUTAMMATE_DOCKING_SITE.mp4** — Gly-146 enlarges the pocket within the super-complex; ions visualise the broadened basin.
- **Cacng8_val146gly_psd93_95_cacng2_7_ppp3ca_cb_r1_cnih2_3_GLUTAMMATE.mp4** — Egress path shows an expanded vestibular funnel guided by scattered ion sites.
- **Cacng8_arg123ter_psd93_95_cacng2_7_ppp3ca_cb_r1_cnih2_3_DOCKING_SITE.mp4** — Docked glutamate reveals a re-engineered pocket; ions highlight the modified niche.
- **Cacng8_arg123ter_psd93_95_cacng2_7_ppp3ca_cb_r1_cnih2_3_GLUTAMMATE.mp4** — Egress route illustrating charge-guided diffusion in the truncation mutant.
- **Cacng8_arg123ter_psd93_95_cacng2_7_ppp3ca_cb_r1_cnih2_3_MD.mp4** — 100 ns MD shows scaffold-modulated gating with stable ligand binding.
- **Cacng8_leu96val_arg123ter_psd93_95_cacng2_7_ppp3ca_cb_r1_cnih2_3_GLUTAMMATE_DOCKING_SITE.mp4** — Docked glutamate in the double mutant super-complex; ions expose combined electrostatic alterations.
- **Cacng8_leu96val_arg123ter_psd93_95_cacng2_7_ppp3ca_cb_r1_cnih2_3_GLUTAMMATE.mp4** — Ligand egress shows synergistic changes to the exit corridor created by both substitutions.
